# Low-quality employment trajectories and risk of common mental disorders, substance use disorders and suicide attempt: a longitudinal study of the Swedish workforce

**DOI:** 10.5271/sjweh.3978

**Published:** 2021-09-30

**Authors:** Johanna Jonsson, Carles Muntaner, Theo Bodin, Magnus Alderling, Rebeka Balogh, Bo Burström, Letitia Davis, Virginia Gunn, Tomas Hemmingsson, Mireia Julià, Katarina Kjellberg, Bertina Kreshpaj, Cecilia Orellana, Eva Padrosa, David H Wegman, Nuria Matilla-Santander

**Affiliations:** Unit of Occupational Medicine, Institute of Environmental Medicine, Karolinska Institutet, Stockholm, Sweden; Bloomberg Faculty of Nursing and Division of Social and Behavioural Sciences, Dalla Lana School of Public Health, Toronto, Canada; Centre for Occupational and Environmental Medicine, Stockholm Region, Stockholm, Sweden; Interface Demography, Vrije Universiteit Brussel, Brussels, Belgium; Institute for Employment Research, University of Warwick, Coventry, United Kingdom; Centre for Epidemiology and Community Medicine, Stockholm County Council, Stockholm, Sweden; Department of Global Public Health, Equity and Health Policy Research Group, Karolinska Institutet, Stockholm, Sweden; Occupational health consultant, Cambridge, Massachusetts, USA; Lawrence S. Bloomberg Faculty of Nursing, University of Toronto & MAP Centre for Urban Health Solutions, Li Ka Shing Knowledge Institute, St. Michael’s Hospital, Unity Health Toronto, Toronto, Canada; Department of Public Health Sciences, Stockholm University, Sweden; Research Group on Health Inequalities, Environment, and Employment Conditions (GREDS), Department of Political and Social Sciences, Universitat Pompeu Fabra, Barcelona, Spain; Johns Hopkins University – Universitat Pompeu Fabra Public Policy Center, Barcelona, Spain; University of Massachusetts Lowell, Lowell, USA

**Keywords:** alcohol, anxiety, cohort study, depression, drug, employment transition, epidemiology, labor market trajectory, mental health, precarious employment, public health, Sweden

## Abstract

**Objective:**

High-quality longitudinal evidence exploring the mental health risk associated with low-quality employment trajectories is scarce. We therefore aimed to investigate the risk of being diagnosed with common mental disorders, substance use disorders, or suicide attempt according to low-quality employment trajectories.

**Methods:**

A longitudinal register-study based on the working population of Sweden (N=2 743 764). Employment trajectories (2005–2009) characterized by employment quality and pattern (constancy, fluctuation, mobility) were created. Hazard ratios (HR) were estimated using Cox proportional hazards regression models for first incidence (2010–2017) diagnosis of common mental disorders, substance use disorders and suicide attempt as dependent on employment trajectories.

**Results:**

We identified 21 employment trajectories, 10 of which were low quality (21%). With the exception of constant solo self-employment, there was an increased risk of common mental disorders (HR 1.07–1.62) and substance use disorders (HR 1.05–2.19) for all low-quality trajectories. Constant solo self-employment increased the risk for substance use disorders among women, while it reduced the risk of both disorders for men. Half of the low-quality trajectories were associated with a risk increase of suicide attempt (HR 1.08–1.76).

**Conclusions:**

Low-quality employment trajectories represent risk factors for mental disorders and suicide attempt in Sweden, and there might be differential effects according to sex – especially in terms of self-employment. Policies ensuring and maintaining high-quality employment characteristics over time are imperative. Similar prospective studies are needed, also in other contexts, which cover the effects of the Covid-19 pandemic as well as the mechanisms linking employment trajectories with mental health.

Mental disorders are among the leading disease burdens in middle- and high-income countries such as Sweden (1). The indirect costs of mental disorders (ie, days of sick leave) have increased in recent years (2). Moreover, mental disorders are unequally distributed, being substantially more present among individuals in lower socioeconomic positions (3). It is thus imperative to analyze the causes of mental disorders to adopt appropriate preventive and health promotion policies. In this paper, we focus on low-quality employment through a multidimensional conceptualization and operationalization of precarious employment (PE), characterized by employment insecurity, income inadequacy and lack of rights and protection, features commonly included in PE (4). PE is a well-known social determinant of health and health inequalities (5) associated with poor mental health among workers all over the world (6).

Even in countries like Sweden – with strong and influential unions, high rates of collective bargaining agreement coverage and employment protection – PE is cause for concern. The proportions of unemployment as well as temporary employment have been relatively stable since the dramatic increases after the deep recession of 1990. However, in association with more relaxed employment protection legislation on temporary employment, its most precarious form, on-call employment, has continuously increased and replaced the more secure form of substitution positions. Since 2000, temporary and particularly on-call employment has grown in the lowest occupational wage strata, indicating that the lower end of the occupational structure is becoming more precarious (7).

To date, there are several knowledge gaps in terms of PE’s relationship with poor mental health. For one, the association has largely been explored in studies using unidimensional measures of PE and/or a cross-sectional design. Indeed, a recent metanalysis of longitudinal studies found inconclusive results associated with a scarcity of high-quality studies and a lack of comparability between them (6). Furthermore, most studies do not adequately address reversed and bi-directional causality and rely heavily on self-reported data, which could lead to overestimation of associations. Additionally, an individual’s working life course is complex (8) involving states with various combinations of employment conditions (characterized by lower or higher quality) as well as transitions between these, which could have further health implications. For example, employment trajectories characterized by upshifting from lower to higher employment quality or vice versa (upwards or downwards mobility), or alternating employment with unemployment (fluctuations), have shown associations with mental health (9, 10). However, although previous literature on trajectories and mental health generally is limited, there is a particular scarcity of studies assessing trajectories characterized by fluctuating patterns as well as outcomes of suicide attempt/suicide or abuse of non-alcoholic substances.

Furthermore, certain workforce subgroups are important to include and analyze properly when studying low-quality employment and health. Trends in precarious forms of self-employment (what ILO calls disguised employment relationships) such as those associated with the gig economy and/or bogus self-employment (workers falsely classified as self-employed by employers to avoid employment taxes) are increasing, in the EU as well as in Sweden (11). Low-quality self-employment has been associated with poor mental health in previous European studies (12, 13), making this an important group to study. Consideration also needs to be given to unemployment, a common occurrence for many workers in low-quality employment. A few published studies suggest that individuals who transition from PE to unemployment or the other way around, have higher odds of depression [eg, (14)]. Moreover, many labor markets today – including the Swedish one – are gender segregated (women and men have different occupational positions and are in different spheres of the labor market) (15), which necessitates a gender-stratified approach.

Data-driven approaches such as latent class analysis (LCA) allows for the creation of employment typologies, ie, explorations of how employment conditions such as income level, temporariness, etc, cluster based on similarity. In Sweden (16) and Europe (12, 13), previous applications of LCA have resulted in numerous employment types with distinct features as well as indications of lower and higher quality. Applying LCA across time in an inclusive population (including salaried, self-employed, and unemployed individuals), not only as a total population but also separately for women and men, could serve as means to study more complex labor market trajectories and further our understanding of the effects of low-quality employment trajectories on mental health.

Consequently, our objective was to investigate the risk of being diagnosed with a common mental disorder, substance use disorder or suicide attempt according to low-quality employment trajectories. We expect that individuals in low-quality employment trajectories characterized by constancy (remaining in constant low-quality employment), fluctuation (moving in-and-out of low-quality, or in-and-out of low- and high-quality employment), or directional mobility (moving from one low-quality employment to another, moving from high- to low-quality employment) are at increased risk of poor mental health.

## Methods

### Study design, setting and data collection

This is a longitudinal register-study based on the Swedish Work, Illness, and Labour-market Participation (SWIP) cohort (16). The SWIP cohort is the result of linkage of multiple registers and includes all registered individuals in Sweden, aged 16–65 years (approximately 5.4 million individuals), in 2005 and followed until 2017. The present study uses a subpopulation of 2 743 764 individuals aged 18–61 years old residing in Sweden in 2005. For this cohort, employment trajectories were created for 2005–2009 and mental disorder diagnoses followed up between 2010–2017. Exclusion criteria for the study were: (i) incomplete information for measuring the exposure variable (2005–2009), (ii) yearly employer-based income of >100 SEK, (iii) death, emigration, or immigration during 2005–2009, (iv) any mental disorder diagnosis (ICD-10 codes: F00-F99) or suicide attempt (ICD-10: X60-X84 and Y10-Y34) occurring between 2003–2009 (ie, two years preceding, or occurring during, exposure assessment) in order to minimize risk of reverse causation. See flow chart of the total population in the supplementary material (https://www.sjweh.fi/article/3978) figure S1.

Sociodemographic and employment data were collected for 2003–2009 from the Longitudinal Integration Database for Health Insurance and Labor Market Studies register (LISA) (18). Date of diagnosis in inpatient and specialized outpatient care, serving as the outcome, was retrieved from the National Patient Register (NPR) for 1964–2017 (19). Linkage with parental data (both biological and adoptive) was conducted by means of the multi-generational register. Data on parental history of mental disorders were retrieved from the NPR (using inpatient data only), while socioeconomic data were retrieved from the population and housing censuses of 1960, 1970, 1980 and 1990. Statistics Sweden linked all data sources.

The Regional Ethics Board of Stockholm granted ethical permission for the study (no. 2017/1224-31/2 and 2018/1675-32).

### Exposure assessment: Employment trajectories

Employment trajectories spanning 2005 to 2009, characterized by employment quality and pattern (constancy, fluctuation, direction of mobility), were created for the total population, as well as for women and men separately. This was done in two steps, as briefly described below. Complete details are provided in the supplementary material.

### Step 1: Creating an employment typology

In order to extract a class-solution (employment typology) applicable to the entire period of 2005–2009, the Swedish Register-based Operationalization of Precarious Employment (SWE-ROPE) (16) was implemented for each year and thereafter a number of repeated measures latent class analyses (RMLCA) were performed. Briefly, SWE-ROPE includes the following dimensions and items: (i) employment insecurity, including contractual relationship insecurity (employment contract with employer or other party), contractual temporariness (stable or unstable employment), and multiple jobs and/or in multiple sectors, (ii) income inadequacy, including yearly income level, and (iii) lack of rights and protection, including lack of unionization. SWE-ROPE is based on the employer providing the largest source of income. Further, SWE-ROPE was constructed based on a review of definitions of PE (4) and has previously been used to identify precarious employment/low-quality employment in Sweden (16). The final class-solution contained six classes (employment types). This decision was reached by comparing the three initially best class-solutions in terms of fit indices, classification diagnostics and data-patterns (entropy, average posterior probabilities (AVEPP), comparison of class homogeneity/class separation, and plotting conditional item-probabilities). The fit-indices indicated best fit for the seven-class solution and closely thereafter, the six-class solution, while the Entropy and AVEPP indicated best fit for the five-class solution. The six-class solution, however, showed the most distinct and meaningful classes and was thereby chosen as the final solution. The process was similar in the separate analyses of women and men. The classes were labelled based on information gained from the conditional item-probability plots, class homogeneity and class separation: precarious employment relationship (PER), solo self-employment (SSE), hybrid multiple job-holding (HMJH), business ownership (BO), standard employment relationship (SER), and standard employment relationship with high income (SER/HI). A seventh type, unemployment (UE), was added separately. This group was defined by unemployment >180 days during the year and having information on at least one employer (ie, they were still in the workforce). Fit statistics, characteristics of the employment typology, and conditional item-probabilities can be found in supplementary tables S1, S2 and S3 respectively.

### Step 2: Creating employment trajectories

Employment trajectories were created by combining the employment types and UE (jointly referred to as employment states) across the five years. Trajectories were manually created to enable categorization of individuals in patterns of (i) constancy (spending 4–5 years in an employment state), (ii) fluctuation (spending ≥2 years in two employment states with fluctuating movement in-and-out between these), and (iii) direction of mobility (spending ≥1 year in an employment state at the beginning of the trajectory, and ≥2 years in another state at the end of the trajectory). If trajectories matched several patterns, grouping was done in the following hierarchy: fluctuation, constancy, mobility. The 8230 initial combinations were first reduced to 68 trajectories and, thereafter, further to 21 trajectories. In this process, for analytical feasibility, employment states were merged according to their perceived employment quality: (i) low (PER, SSE, UE), (ii) high (SER, SER/HI, BO) and (iii) HMJH, which could not be determined to be either high or low quality in this study and hence kept separate. Similar interpretation of the quality of the employment types has been made in a previous study (16). Once the 21 trajectories were created, trajectories of low quality were identified as based on (i) the quality of employment states included in the trajectory, ie, the presence of low-quality employment states, and (ii) the pattern of the trajectory, ie, the direction could not entail an improvement in conditions across the trajectory (upwards mobility). As HMJH could be considered neither low nor high quality, all trajectories characterized by low-quality employment states and HMJH were considered to be low quality. See table 1 for details on how specific combinations of employment states were grouped under the patterns, and supplementary table S4 for details on the reduction of 68 to 21 trajectories.

**Table 1:** Description of employment trajectories identified in the cohort. [UE=unemployment; PER=precarious employment relationship; SSE=solo self-employment; HMJH=hybrid multiple job-holding; BO= business ownership; SER= standard employment relationship; SER/HI=standard employment relationship with high income; LQ=low quality; HQ=high quality].

**Constant trajectories**	
Classification: Spending 4-5 years in a specific employment state, or in combinations of LQ (UE, PER, SSE) or HQ (BO, SER, SER/HI) employment states, independent of order through time. See examples under each specific trajectory.	
SER	Spending 4-5 years in SER. Example: SER-SER-PER-SER-SER, SER-SER-SER-SER-SER
SER/HI	Spending 4-5 years in SER/HI. Example: SER/HI-SER-SER/HI-SER/HI-SER/HI, SER/HI-SER/HI-SER/HI-SER/HI-SER/HI
BO	Spending 4-5 years in BO. Example: BO-BO-BO-BO-BO, BO-HMJH-BO-BO-BO
HMJH	Spending 4-5 years in HMJH. Example: HMJH-HMJH-HMJH-HMJH-PER, HMJH-HMJH-BO-HMJH-HMJH
SSE a	Spending 4-5 years in SSE. Example: SSE-SSE-SSE-SSE-PER, SSE-SSE-SSE-SSE-SSE
PER a	Spending 4-5 years in PER. Example: UE-PER-PER-PER-PER, PER-PER-PER-PER-PER
UE a	Spending 4-5 years in UE. Example: UE-UE-HMJH-UE-UE, UE-UE-UE-UE-UE
HQ	Spending 4-5 years in any other combination of HQ employment states (BO, SER, SER-high income). Example: BO-HMJH-SER-SER/HI-BO, SER-SER-SER/HI-SER/HI-BO
LQ a	Spending 4-5 years in any other combination of LQ employment states (UE, PER, SSE). Example: PER-SSE-HMJH-UE-PER, UE-PER-PER-PER-HMJH, PER-PER-PER-SSE-UE
**Fluctuating trajectories**	
Classification: Spending a minimum of two years in two employment states with fluctuating movement in-and-out of the these. Combinations of LQ- and HQ-employment states are also included (if a minimum of two years in the LQ- and HQ-classes, respectively). See examples under each specific trajectory.	
HQ	Fluctuating movement in-and-out of BO and SER, BO and SER/HI or SER and SER/HI. Example: UE-SER-SER/HI-SER-SER/HI, BO-BO-SER/HI-BO-SER/HI
LQ a	Fluctuating movement in-and-out of UE and PER, UE and SSE, or PER and SSE. Example: PER-SE-SE-PER-SER, UE-PER-UE-PER-PER
HQ and HMJH	Fluctuating movement in-and-out of BO and HMJH, SER and HMJH, SER/HI and HMJH, or any other combination of HQ employment states and HMJH. Example: SER-HMJH-HMJH-SER/HI-SER, HMJH-BO-HMJH-BO-SSE, SER-SER-HMJH-HMJH-SER/HI
LQ and HMJH a	Fluctuating movement in-and-out of UE and HMJH, PER and HMJH, SSE and HMJH, or any other combination of LQ employment states and HMJH. Example: PER-HMJH-HMJH-UE-PER, SER/HI-PER-HMJH-PER-HMJH, SSE-UE-HMJH-HMJH-PER
LQ and HQ a	Fluctuating movement in-and-out of UE and BO, UE and SER, UE and SER/HI, PER and BO, PER and SER, PER and SER/HI, SSE and BO, SSE and SER, SSE and SER/HI, or other combinations LQ- and HQ-employment states. Example: BO-SER-PER-SER-PER, SSE-BO-BO-SSE-UE, PER-PER-BO-PER-SER
**Trajectories characterized by mobility**	
Classification: Spending a minimum of one year in one employment state at the beginning of the trajectory, followed by a minimum of two years in another employment state at the end of the trajectory. The last two years could also be characterized by combinations of LQ- or HQ-employment states. Mobility can be upwards, downwards, within or between. See examples under each specific trajectory.	
Within HQ	Moving from one HQ state to another HQ state, i.e., from BO to SER or SER/HI, from SER to SER/HI or BO, or from SER/HI to SER or BO. Example: SER/HI-BO-HMJH-SER-SER
Between HMJH and HQ	Moving between a HQ state and HMJH or the other way around, i.e., between BO, SER or SER/HI and HMJH, or between HMJH and BO, SER or SER/HI. Example: SER-SER/HI-BO-HMJH-HMJH, BO-BO-HMJH-HMJH-UE, HMJH-HMJH-SER/HI-SER/HI-SER/HI, HMJH-PER-UE-SER-SER
Between HMJH and LQ a	Moving between a LQ state and HMJH or the other way around, i.e., between UE, PER or SSE and HMJH, or HMJH and UE, PER or SSE. Example: PER-SSE-HMJH-HMJH-HMJH, UE-UE-SER-HMJH-HMJH, HMJH-SER-SER-UE-SSE, HMJH-HMJH-SSE-PER-PER
Within LQ a	Moving from one LQ state to another LQ state, i.e., from UE to PER or SSE, from PER to SSE or UE, or from SSE to UE or PER. Example: PER-SER-HMJH-UE-UE, UE-SER-SER-SSE-SSE
Upwards LQ to HQ	Moving from UE, PER, SSE to BO, SER, SER/HI or a combination of HQ employment states. Example: PER-PER-SER-SER/HI-SER/HI, SSE-HMJH-HMJH-BO-BO, PER-SSE-SER-SER-SER/HI
Downwards HQ to LQ a	Moving from BO, SER or SER/HI to UE, PER, SSE or a combination of LQ employment states. Example: BO-BO-HMJH-PER-PER, SER-SER/HI-BO-SSE-SSE, SER-PER-HMJH-PER-SSE
**Other trajectories**	
Classification: Any other combination of employment states, UE, PER, SSE, BO, SER, SER/HI, not following any clear pattern and not otherwise classified	

a Low-quality employment trajectories (according to the a priori hypotheses of the study).

### Outcome assessment: diagnosis of common mental disorders, substance use disorders and suicide attempt

First incidence of diagnosis occurring 2010–2017 was identified in the NPR by the following ICD-10 codes: (i) common mental disorders – depression (F32-F33), anxiety (F41) and stress-related disorders (F43); substance use disorders – alcohol (F10), opioids (F11), cannabinoids (F12), sedatives or hypnotics (F13), cocaine (F14), other stimulants including caffeine (F15), hallucinogens (F16), volatile solvents (F18), and multiple drug use and other psychoactive substances (F19); (iii) suicide attempt – intentional self-harm (X60-X84) and events of undetermined intent (Y10-Y34) (20).

### Potential confounders

We obtained the minimal sufficient set of variables for adjustment by drawing the causal assumptions in a directed acyclic graph (DAG) (supplementary figure S2). Confounders included: (i) age, highest completed education (primary and secondary school; tertiary education <3 years; tertiary education ≥3 years), country of birth (Sweden; within EU-28; outside EU-28), and marital status (married/cohabiting with children; married/cohabiting without children; single with children; single without children). These were measured in 2005 (ie, the start of the exposure assessment); (ii) individual history of any mental disorder diagnosis (ICD-10: F00-F99; ICD-9: 290-316; ICD-8: 290-309) or suicide attempt (ICD-10: X60-X84, Y10-Y34; ICD-9: E950-E959, E980-E989; ICD-8: E950-E959 (21) (yes; no) registered in inpatient care between 1964-2002 (before exposure measurement and exclusion criteria); and (iii) parental history of any mental disorder diagnosis or suicide attempt, using the same ICD-codes as above (more than one parent; no parent), between 1964-2004 (before exposure assessment). Parental socioeconomic position in the individual’s childhood (manual; non-manual; farmer/self-employed), measured in 1960, 1970, 1980 or 1990. Missing data on any of these confounders was categorized as “unknown”.

### Statistical analysis

Cox proportional hazard regression models were applied to estimate hazard ratios (HR) with 95% confidence intervals (CI) for the outcomes as dependent on employment trajectories. The trajectory “constant SER” was used as reference in all models. Person-time was calculated from the 1 January 2010, until first incidence of diagnosis, end of follow-up (31 December 2017), or until censoring due to death or emigration, whichever came first. The time-lag between the exposure and outcome assessment was chosen for capturing short-, mid- and long-term effects of the trajectories. One un­adjusted model and two confounder-adjusted models were run for each outcome. The first adjusted model included individual characteristics and the second model both individual and parental characteristics. All analyses were conducted for the total population and women and men separately. Furthermore, additional analyses were performed to explore the effect of employment trajectories on five specific outcomes: depression (ICD-10 codes F32-F33), anxiety (F41), stress-related disorders (F43), alcohol disorders (F10), and other drug-related disorders (F11-F16, F18-F19). The RLMCA were run in Mplus version 8.4 (Muthén & Muthén, Los Angeles, CA, USA), while remaining data management and statistical analysis was conducted with SAS version 9.4.0 (SAS Institute Inc, Cary, NC, USA) and STATA version 16 (StataCorp LLC, College Station, TX, USA). Figures were produced in RStudio version 1.2.5033 (RStudio, PBC, Boston, MA, USA).

## Results

We found six employment types in the total population and among women and men. Women and men had similar-sized types of PER and HMJH, whereas most women were in SER and the majority of men in SER/HI. Further, both SSE and BO were more common among men. Women generally had lower income than men across all employment types, although particularly evident in the low-quality employment types of PER and SSE (supplementary table S3).

Combining the employment types and UE resulted in 21 employment trajectories (table 1), 10 of which were considered low-quality (4 constant, 3 fluctuating, 3 of mobility), accounting for 20.6% of the cohort. Table 2 shows the characteristics of individuals in each trajectory (for full characteristics see supplementary table S5). Generally, individuals in low-quality trajectories were less often highly educated, born in Sweden and married/cohabiting with a partner, while they more often had a history of mental disorders prior to 2003, as compared with individuals in high-quality trajectories. Young individuals were over-represented in constant PER. Trajectories of women and men showed similar patterns across individual characteristics (supplementary table S6a–b).

**Table 2:** Characteristics of cohort (2009) according to employment trajectories. [UE=unemployment; PER=precarious employment relationship; SSE=solo self-employment; HMJH=hybrid multiple job-holding; BO= business ownership; SER= standard employment relationship; SER/HI=standard employment relationship with high income; LQ=low quality; HQ=high quality].

Trajectories	Total		Women	Age	Higher level of education a	Born in Sweden	Married/cohabiting	Mental disorder before 2003
						
	N	%	%	Mean	%	%	%	%
Total	2 743 764	100	46.5	46.5	37.7	90.6	66.6	2.8
Constant								
SER	1 049 775	38.3	64.5	47.7	28.9	89.7	66.1	3.2
SER/HI	597 315	21.8	27.8	48.6	60.8	92.5	71.5	1.8
BO	45 587	1.7	15.7	49.2	24.7	93.7	78.2	1.7
HMJH	43 437	1.6	35.1	48.0	40.5	94.3	69.7	2.2
SSE b	80 786	2.9	27.9	50.7	21.8	88.2	68.5	2.5
PER b	103 578	3.8	46.9	38.5	29.2	90.9	53.9	3.4
UE b	1459	0.05	32.4	46.8	24.7	72.2	44.6	6.8
HQ	133 239	4.9	37.9	45.5	42.5	90.9	67.2	2.4
LQ b	16 954	0.6	34.3	42.7	25.5	83.6	56.2	4.2
Fluctuating								
HQ	64 817	2.4	35.3	46.1	39.9	90.6	67.5	2.7
LQ b	1990	0.07	32.0	43.1	22.9	80.1	48.6	5.7
HQ and HMJH	30 086	1.10	33.1	46.9	41.4	94.3	28.9	2.1
LQ and HMJH b	13 815	0.5	32.4	44.7	35.1	90.0	63.1	2.8
LQ and HQ b	217 745	7.9	43.8	42.7	31.1	89.5	37.8	3.0
Mobility								
Within HQ	5620	0.2	33.6	45.7	32.5	91.2	66.2	2.7
Between HMJH and HQ	37 998	1.4	38.0	46.2	41.2	93.5	69.7	2.3
Between HMJH and LQ b	15 650	0.6	35.2	44.8	35.8	91.1	64.0	2.7
Within LQ b	575	0.02	31.1	43.8	24.9	82.4	54.6	4.0
Upwards LQ to HQ	126 716	4.6	48.3	42.0	33.2	90.2	61.7	3.2
Downwards HQ to LQ b	116 575	4.2	44.1	43.4	31.6	89.5	62.4	3.1
Other	40 047	1.5	32.3	43.7	37.1	90.8	66.0	2.7

a Higher level of education: includes tertiary education of more or less than 3 years and is compared to lower level of education (elementary education of less than or equal to 12 years).

b Low-quality employment trajectories (according to the a priori hypotheses of the study).

Individuals in low-quality employment trajectories generally had higher incidence of common mental disorders, substance use disorders, and suicide attempt, compared with high-quality employment trajectories, both overall and among women and men respectively (see table 3). The exception was constant SSE, which generally showed a low incidence across all outcomes. Furthermore, the incidence of common mental disorders was overall higher for women compared to men, while it was the reverse for substance use disorders and suicide attempt.

**Table 3:** Cumulative incidence (I) of common mental disorders, substance use disorders and suicide attempt per 100 persons (2010–2017) according to employment trajectories (2005–2009). [UE=unemployment; PER=precarious employment relationship; SSE=solo self-employment; HMJH=hybrid multiple job-holding; BO= business ownership; SER= standard employment relationship; SER/HI=standard employment relationship with high income; LQ=low quality; HQ=high quality].

Trajectories	Common mental disorders						Substance abuse						Suicide attempt					
		
	Total N=2 743 764		Women N=1 275 850		Men N=1 467 914		Total N= 2 743 764		Women N=1 275 850		Men N=1 46 7914		Total N= 2 743 764		Women N=1 275 850		Men N=1 467 914	
								
	Cases	I	Cases	I	Cases	I	Cases	I	Cases	I	Cases	I	Cases	I	Cases	I	Cases	I
Constant																		
SER	40 261	3.8	27 645	4.1	12 100	3.3	12 370	1.2	5245	0.8	6902	1.9	6350	0.6	3659	0.5	2606	0.7
SER/HI	14 450	2.4	5446	3.3	8831	2.1	5942	1.0	993	0.6	4886	1.1	2864	0.5	708	0.4	2124	0.5
BO	1088	2.4	282	3.2	962	2.2	605	1.3	80	0.9	620	1.4	267	0.6	43	0.5	257	0.6
HMJH	1361	3.1	803	4.3	642	2.6	448	1.0	136	0.7	303	1.2	255	0.6	93	0.5	143	0.6
SSE a	2362	2.9	879	3.9	1672	2.6	1133	1.4	214	1.0	1013	1.6	474	0.6	120	0.5	410	0.6
PER a	5808	5.6	3438	7.0	2389	4.3	1705	1.6	521	1.1	1189	2.2	902	0.9	345	0.7	562	1.0
UE a	97	6.6	69	7.2	121	4.9	60	4.1	8	0.8	97	3.9	20	1.4	5	0.5	23	0.9
HQ	4439	3.3	2124	4.2	2318	2.8	1549	1.2	377	0.7	1176	1.4	829	0.6	263	0.5	561	0.7
LQ a	915	5.4	518	6.8	599	4.6	394	2.3	109	1.4	362	2.8	160	0.9	50	0.7	144	1.1
Fluctuating																		
HQ	2079	3.2	967	4.1	1079	2.7	829	1.3	174	0.7	638	1.6	387	0.6	128	0.5	256	0.6
LQ a	134	6.7	64	7.7	86	5.5	65	3.3	15	1.8	52	3.3	19	1.0	6	0.7	17	1.1
HQ and HMJH	906	3.0	438	3.9	455	2.5	296	1.0	75	0.7	206	1.1	198	0.7	61	0.6	123	0.7
LQ and HMJH a	604	4.4	264	5.8	335	3.4	213	1.5	45	1.0	168	1.7	109	0.8	32	0.7	81	0.8
LQ and HQ a	9258	4.2	5038	5.3	4222	3.4	2948	1.3	806	0.8	2155	1.7	1454	0.7	562	0.6	900	0.7
Mobility																		
Within HQ	206	3.7	92	4.8	93	3.3	80	1.4	18	0.9	52	1.9	35	0.6	12	0.6	18	0.6
Between HMJH and HQ	1240	3.3	671	4.4	588	2.6	357	0.9	94	0.6	266	1.2	228	0.6	85	0.6	146	0.6
Between HMJH and LQ a	653	4.2	334	5.9	337	3.3	196	1.3	49	0.9	157	1.5	93	0.6	31	0.5	73	0.7
Within LQ a	37	6.4	13	7.5	25	6.3	14	2.4	2	1.1	12	3.0	5	0.9	2	1.1	3	0.8
Upwards LQ to HQ	5438	4.3	3166	5.2	2218	3.4	1660	1.3	540	0.9	1102	1.7	826	0.7	326	0.5	494	0.8
Downwards HQ to LQ a	5030	4.3	2796	5.5	2237	3.4	1792	1.5	524	1.0	1291	1.9	833	0.7	355	0.7	487	0.7
Other	1627	4.1	726	5.6	911	3.3	501	1.3	103	0.8	382	1.4	264	0.7	72	0.6	186	0.7
Total	97 993	3.6	55 773	4.4	42 220	2.9	33 157	1.2	10 128	0.8	23 029	1.6	16 572	0.6	6958	0.5	9614	0.7

a Low-quality employment trajectories (according to the a priori hypotheses of the study).

### Common mental disorders

**Figure 1a–c:**
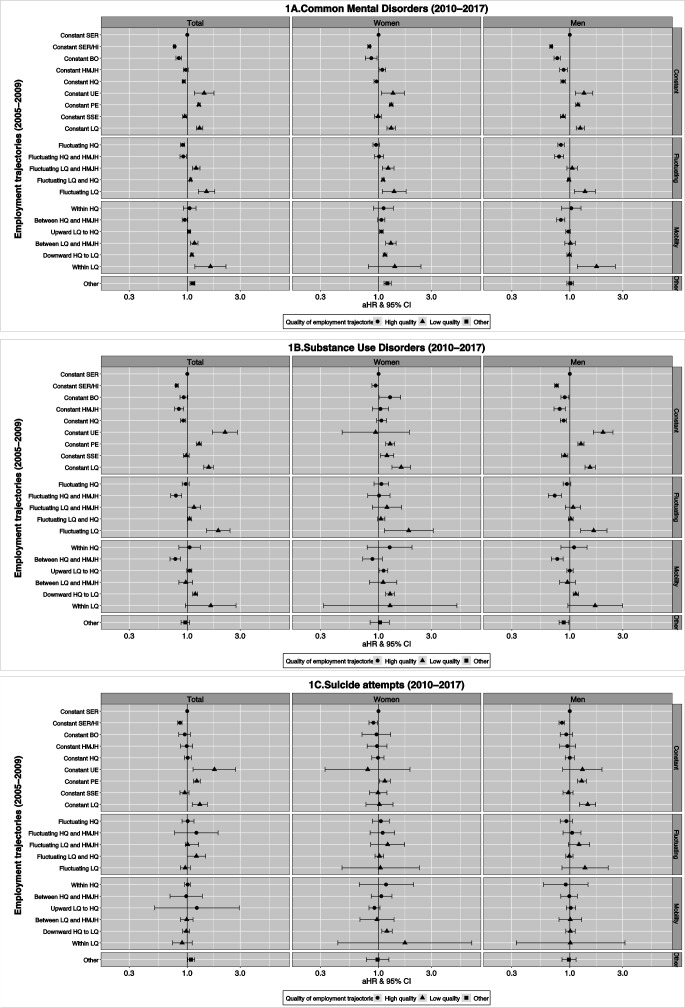
Adjusted hazard ratios (aHR) and their 95% confidence intervals (95%CI) of common mental disorders, substance use disorders and suicide attempts (2010-2017) according to employment trajectories (2005-2009). aHR adjusted for sex, age in 2005, level of education in 2005, marital status in 2005, country of birth, any mental disorder before 2003, parental mental disorders, parental socioeconomic position during childhood. Total (N=2 743 764), men (N=1 467 914), women (N=1 275 850). [UE=unemployment; PER=precarious employment relationship; SSE=solo self-employment; HMJH=hybrid multiple job-holding; BO= business ownership; SER= standard employment relationship; SER/HI=standard employment relationship with high income; LQ=low quality; HQ=high quality].

The risk of being diagnosed with a common mental disorder was increased among individuals in all low-quality employment trajectories, as compared with individuals in constant SER (fully adjusted HR 1.07–1.62), except for constant SSE (HR 0.95). Mobility within low-quality employment, fluctuating low-quality employment and constant UE showed the largest risk estimates (HR 1.62, 1.49, and 1.42, respectively, although with wide 95% CI). In addition to constant SSE, constant SER/HI and BO were also associated with reduced risk (HR 0.77 and 0.84, respectively). Men generally had smaller risk estimates compared to women in these trajectories. Additional differences were seen in terms of constant HMJH (increased risk for women, reduced risk for men), mobility between HMJH and low-quality employment, fluctuation in-and-out of low-quality and HMJH (both showed increased risk for women only), and constant SSE (reduced risk for men only). See figure 1 for fully adjusted estimates and table S7a for all estimates. Estimates for depression, anxiety, and stress related disorders separately showed similar patterns (table S8a–c for all, women, and men, respectively).

### Substance use disorders

Low-quality employment trajectories were associated with an increased risk of substance use disorders (HR 1.05–2.19), with the exceptions of constant SSE and mobility between HMJH and low-quality employment. The largest risk estimates were found for constant UE (HR 2.19), fluctuating low-quality employment (HR 1.90) and constant low-quality employment (HR 1.56). Reduced risk was seen among individuals in the constant trajectories of high-quality employment (HR 0.92), HMJH (HR 0.84) and SER/HI (HR: 0.80). However, in the sex-specific analyses, these trajectories were associated with reduced risk in men only. Furthermore, reduced risk was found for men in constant SSE and BO (HR 0.90 and 0.90, respectively), while the risk was increased among women in these trajectories (HR 1.19 and 1.27). See figure 1 for adjusted estimates and supplementary table S7b for all estimates. Estimates for alcoholic and non-alcoholic substance use disorders, respectively, showed similar patterns (supplementary table S8a-c).

### Suicide attempt

The risk of suicide attempt was increased for individuals in half of the low-quality trajectories, including constant UE (HR 1.76), low-quality (HR 1.30), PER (HR 1.22), fluctuation in-and-out of low-quality and HMJH (HR 1.21), and downwards mobility from high-quality to low-quality (HR 1.08). Reduced risk was seen for individuals in constant SER/HI (HR 0.86), effects which remained for both women and men. However, constant low-quality and fluctuation in-and-out of low-quality and HMJH was only associated with an increased risk among men, and mobility from high-quality to low-quality only among women. See figure 1 for adjusted estimates and table S7c for all estimates.

## Discussion

### Main findings

This study identified 21 employment trajectories across five years in the Swedish labor market. Ten of these trajectories were considered low-quality, accounting for 21% of the cohort. With the exception of constant SSE, all low-quality employment trajectories increased the risk of diagnosis of common mental disorder and substance use disorder. Half of the low-quality trajectories were associated with an increased risk of suicide attempt. The constant trajectories PER, UE and low-quality employment were risk factors for all three outcomes.

Contrary to our expectations, only women in constant SSE showed an increased risk of substance use disorders, while there was a tendency for reduced risk in the total population and among men in terms of both substance use and common mental disorders. Women also had increased risk of common mental disorders in trajectories characterized by low-quality employment and HMJH, which men did not.

### Interpretation

Overall, individuals who experienced low-quality employment trajectories – characterized by patterns of constancy, fluctuation, or mobility – had an increased risk of common mental disorders, substance use disorders and suicide attempt. These findings are in line with the expectations of the study, as well as with previous studies exploring the relationship between employment trajectories and mental health using various measures of employment quality and labor market position (14, 22–25).

In terms of low-quality employment trajectories characterized by fluctuations and mobility, previous Swedish and Swiss studies have found unstable trajectories characterized by multiple transitions to be associated with increased risk of receiving a psychiatric diagnosis (22) and depressive symptoms (24). Further, within low-quality mobility has been found to be associated with an increased risk of psychological distress (10) and downwards mobility with poor mental health (25). Estimates in our study, however, must be interpreted with caution due to the low sample size in some of these trajectories. The harmful effects of these trajectory patterns should therefore be explored in future studies, both quantitative and qualitative, the latter of which could also shed light on potential mechanisms involved.

Apart from constant SSE, constant low-quality trajectories were risk factors for all outcomes. The association between trajectories of constant low-quality positions, characterized by poor labor market attachment (ie, non-standard employment, unemployment, PE) and long-term difficulties, and poor mental health has been shown in a few previous studies (14, 22, 26). Contrary to expectation, only constant SSE among women was associated with an increased risk of substance use disorders, while men in both constant SSE and constant BO showed a decreased risk of substance use disorders and common mental disorders. Previous cross-sectional studies conducted in Europe showed that some types of low-quality self-employment, such as dependent self-employment, increase the likelihood of poor mental well-being (12, 13). Unfortunately, no sex-specific results were provided in these studies, which hinders comparison with our results. Our results could be explained by several reasons. First, self-employed women in our study had lower income than men, which is known to have effects on substance use disorders (27). Second, self-employed women generally report high job demands (ie, long working hours, tight deadlines) (28), which could affect the work-life balance of women more negatively than of men given that women still take on more responsibility for household and childcare (29), and further affect mental health. Indeed, self-employed women and men tend to distribute time in a more gender-traditional manner (30), and self-employed men report better work-life balance and well-being compared with both salaried men and self-employed women (28). This could partly explain the reduced effects seen among men in SSE and BO in our study. Third, the data available in our study did not allow to differentiate various forms of solo self-employment, which may imply differential mental effects. Another noteworthy finding in our study is that women belonging to trajectories characterized by HMJH (ie, combination of employment with self-employment) generally had an increased risk of common mental disorders, while men had a decreased risk if in constant HMJH or trajectories characterized by HMJH and high-quality employment. A similar reasoning as with self-employed women and men could be applied to these results. Unfortunately, we do not know the employment relations and conditions of the additional jobs, whether they occurred simultaneously or not, or the reasons for taking on additional jobs, which remains to be explored in future studies. Further, this study found that constant SER/HI entailed a reduced risk for all outcomes, results which support previous reports of eg, poorer mental health outcomes of individuals in discontinuous trajectories as compared with stable trajectories (14, 24, 31). Individuals in SER/HI were characterized by high incomes, stable employment, one job and a high degree of collective bargaining agreement coverage. These results bear important policy health implications, as they point towards that interventions aimed at ensuring and maintaining stable high-quality employment characteristics over time (at least five years) could protect workers from suffering severe common mental disorders in Sweden.

Among possible mechanisms linking low-quality employment trajectories and poor mental health, we note an accumulation of employment, economic and social factors. Factors include employment strain (characterized by the repeated effort of finding employment or a better-quality employment arrangement), job insecurity and other psychosocial exposures in the workplace; income uncertainty and income instability, which may lead to material deprivation as well as have an impact on other social determinants of health (eg, neighborhood quality, lifestyle factors); feelings of precariousness or failure, and social exclusion (5, 24, 32–34). Interestingly, the observed increased risk of mental disorders among individuals following low-quality employment trajectories bear some resemblance with the “deaths of despair” (substance use disorders and suicides caused by poor long-term social and economic outlook) observed during the last decade in the US and the UK (35). Although the constant low-quality trajectories showed clear risk increases in mental disorders, estimates varied across the fluctuating and mobility trajectories. However, the small numbers in some of the trajectories hampers conclusions to be drawn of cross-comparisons of estimates between trajectory patterns. Too little is known about the mechanisms linking specific trajectory patterns and mental disorders, but it is likely that the above-mentioned mechanisms are present (but perhaps to a varying degree) in all these associations.

### Strengths and limitations

This study is based on population registers with high validity and low attrition rates (18), allowing for an objective operationalization of a multidimensional construct of PE. Moreover, the study was based on a large and inclusive (unemployed, self-employed, sex-specific analyses) population, and a longitudinal design. Hence, we believe that the results generally can be extrapolated to the working population of Sweden, and potentially also to other Nordic labor markets with similar welfare regime and labor market legislations. Also, as individuals were without a mental disorder diagnosis at the time of inclusion in the study and during the exposure assessment window, reverse causation is unlikely.

We are, however, only capturing the effect of low-quality employment trajectories on severe mental disorders as we do not have information about visits to primary care, leading to a possible underestimation of the risk estimates. Also, we could not capture the effect of low-quality employment trajectories on actual suicides due to the low number of cases. Future studies using a case–control approach may be able to explore actual suicide. In addition, some trajectories were characterized by small numbers, which implies that the results should be interpreted with caution due to uncertainty of the estimates. Further, the cohort included individuals with exposure information (eg, employer) across all years, which might have led to an exclusion of eg, precarious employees in trajectories of employment and non-employment, leading to an underestimation of effects. Moreover, SWE-ROPE does not as of yet include all items initially described in Kreshpaj et al (4, 16). If all items would have been operationalizable, we may have had a more nuanced employment types and as well as trajectories. This would also have been facilitated by more detailed data as we only had access to data with yearly time-resolution.

### Concluding remarks

By considering both a complex range of characteristics of low-quality employment as well as the dynamic nature of individuals’ working life course, this study adds to the literature an exploration of several low-quality employment trajectories, some of them largely unexplored in previous studies, and their association with mental health. The results demonstrate that low-quality employment trajectories characterized by constancy, fluctuations and mobility indeed are risk factors for severe common mental disorders, substance use disorders and suicide attempt, while the high-quality trajectory of standard employment relationship with high income showed reduced risk. We also found that there could be differential mental health effects according to sex, the most notable being in constant solo self-employment. In the light of these results – as well as the development of temporary employment on the Swedish labor market and the harmful effects of the ongoing COVID-19 pandemic on vulnerable employees – policy interventions aiming at ensuring and maintaining high-quality employment characteristics over time and protecting employees from ending up in unemployment or in otherwise disadvantageous employment trajectories are essential. Similar studies are needed in Sweden as well as in other contexts. In particular, prospective cohort studies covering pandemic effects and mechanisms involved in the relationship between poor employment quality and mental health are needed.

## Supplementary material

Supplementary material
