# Supplementary material for: Low-quality employment trajectories and risk of common mental disorders, substance use disorders and suicide attempt: a longitudinal study of the Swedish workforce
Source: Scand J Work Environ Health. 2021 Sep 30;47(7):509–20. doi: 10.5271/sjweh.3978 (PMC8504160; doi:10.5271/sjweh.3978)
Supplement: Supplementary material [file SJWEH-47-509-S001.pdf]

Low-quality employment trajectories and risk of common mental disorders, substance abuse and suicide attempt: a longitudinal study of the Swedish workforce[1]

by Johanna Jonsson, MSc,[2] Carles Muntaner, PhD, Theo Bodin, PhD, Magnus Alderling, MSc, Rebeka Balogh, MSc, Bo Burström, PhD, Letitia Davis, PhD, Virginia Gunn, PhD, Tomas Hemmingsson, PhD, Mireia Julià, PhD, Katarina Kjellberg, PhD, Bertina Kreshpaj, MSc, Cecilia Orellana, PhD, Eva Padrosa, MSc, David H Wegman, PhD, Nuria Matilla-Santander, PhD

1. *Supplementary material*
2. *Correspondence to: Johanna Jonsson, Unit of Occupational Medicine, The Institute of Environmental Medicine (IMM), Karolinska Institutet, Solnavägen 4, 113 65, Stockholm, Sweden. [E-mail: johanna.jonsson@ki.se]*

INDEX

- Detailed description of the creation of employment trajectories.
- Table S1. Complete and exploratory fit indices for the total population, women and men.
- Table S2. Characteristics of the final employment typology with six classes.
- Table S3. Distribution of conditional item-probabilities for the six-class solution (average across 2005-2009) for the total population, women and men.
- Table S4. Grouping of 68 trajectories into 21 trajectories.
- Table S5. Sociodemographic characteristics of the total population (2005) according to trajectories.
- Table S6a. Sociodemographic characteristics of women according to trajectories.
- Table S6b. Sociodemographic characteristics of men according to trajectories.
- Table S7a. Hazard ratios (crude and adjusted) for common mental disorders for the total population, women and men.
- Table S7b. Hazard ratios (crude and adjusted) for substance use disorders for the total population, women and men.
- Table S7c. Hazard ratios (crude and adjusted) for suicide attempts for the total population, women and men.
- Table S8a. Adjusted hazard ratios for specific outcomes (depression, anxiety, stress related disorders, alcohol and non-alcohol use disorders) for the total population.
- Table S8b. Adjusted hazard ratios for specific outcomes (depression, anxiety, stress related disorders, alcohol and non-alcohol use disorders) for women.
- Table S8c. Adjusted hazard ratios for specific outcomes (depression, anxiety, stress related disorders, alcohol and non-alcohol use disorders) for men.
- Figure S1. Flow chart of the (total) study population.
- Figure S2. Directed Acyclic Graph for the effect of employment trajectories on mental health.

Detailed description of the creation of employment trajectories

Employment trajectories spanning 2005 to 2009, for the total population and for women and men separately, were created in two steps.

First step

Individuals were classified according to employment type (or unemployment) for each year of the exposure assessment window. Individuals that had been unemployed for >180 days during the year were categorized as unemployed. In order to distinguish employment types of precarious nature (or lack thereof) for the non-unemployed population, three dimensions and five items of precarious employment (PE) (as described by Kreshpaj et al 2020), were operationalized for each year by the means of available register data. This process has been thoroughly described in a previous publication (Jonsson et al 2021). Briefly, dimensions, items and their operationalization (within parenthesis) were:

1. *Employment insecurity* with three items, including (1a) contractual relationship insecurity (Directly employed by the employer; Employed by an agency; Combination of self-employed and directly employed; Self-employed with >1 employee; Solo self-employed), (1b) contractual temporariness (Stable employment. i.e., having the same employer for three consecutive years; Unstable employment. i.e., not having the same employer for three consecutive years), and (1c) multiple jobs/sectors (1-2 jobs (employers) during the year; >3 jobs during the year; >3 jobs in >1 economic sector)
2. *Income inadequacy* containing (2a) yearly income level (>200% of the median income of the study population in the specific year; 120-199% of the median; 80-119% of the median; 60-79% of the median; <60% of the median)
3. *Lack of rights and protection* including (3a) lack of unionization (operationalized as likelihood of being covered by collective bargaining agreement in the organization of employment; >90%; 71-90%; 0-70%).

For the sake of the operationalization of contractual temporariness, employer data from LISA was also retrieved for years 2003 and 2004. Thereafter, repeated measures latent class analysis (RMLCA) was performed with the items in order to identify an employment typology and each individual's employment type across the five years. Briefly, RMLCA – being an extension of latent class analysis – is a method aiming at identifying and describing underlying latent classes according to shared features as based on a set of indicators. RMLCA can be applied to longitudinal data as it considers the heterogeneity of the data across time and the emerging latent classes can hence be used to describe change across time (Nylund-Gibson & Choi, 2018). RMLCA produces a set of mutually exclusive and exhaustive classes, estimating the probability of membership in each latent class, and the probability of item-responses'

conditional on each of the latent classes (conditional item-response probabilities) (Collins & Lanza 2009). In RMLCA, the latter two are produced for each year included in the analysis. Class enumeration (i.e., deciding on the number of classes) was done by assessing fit indices, classification diagnostics, and by studying patterns in the data in order to identify a conceptually meaningful class-solution that differentiates between classes (Nylund-Gibson & Choi). The previous included Akaike information criterion (AIC), Bayesian information criterion (BIC) and Sample-size adjusted Bayesian information criterion (SABIC). The two latter included assessing entropy, average posterior probabilities, comparing class homogeneity and class separation, as well as creating and comparing conditional item-probability plots across class-solutions for each PE-item and year. **See fit statistics in table S1.** RMLCA was first applied to half of the data set, i.e., the exploratory data set. Thereafter, the selected class-solution was run on the other half of the data set, i.e., the calibration data set, to validate the model. Finally, as the estimates obtained from the exploratory data set provided an acceptable model fit in the calibration data set, the selected class-solution was run for the complete data set.

Based on a scree plot, the three initially best class-solutions were first selected and thereafter compared. The fit-indices AIC, BIC and SABIC indicated best fit (i.e., produced the smallest numbers) for the seven-class solution and closely thereafter, the six-class solution. The Entropy, however, was highest for the five-cluster solution, and lowest for the six-class solution – although the Entropy of the six-class solution was not much poorer than that of the seven-class solution. The five-class solution also had the highest AVEPP, followed by the six-class solution. In the end, the distinction of the classes in each solution and their meaningfulness (identified by visually inspecting plots of the conditional item-proportions, and in particular by comparing the unique high probabilities, of each solution) led to the decision of the six-class solution as the final solution. In the five-class solution there was no precarious employment relationship-class present, as it emerged first in the six-class solution. In the seven-class solution, however, the business ownership-class split in two (where one class was well off in terms of CBA and income, whereas the other class was not). As we are not investigating, in particular, self-employment, the addition of the second business ownership-class - although interesting - did not provide a strong enough rationale to choose the seven-class solution over the six-class solution. The process was similar in the separate analyses of women and men, although the final six-class solution was chosen after comparing them with four- and five-class solutions, where it was first in the six-class solution that a precarious employment relationship-class emerged. The classes were labelled by information gained from the conditional item-probability plots, class homogeneity and class separation. The six classes identified were: standard employment relationship with high income, standard employment relationship, business ownership, hybrid multiple job-holding, solo self-employment, and precarious

employment relationship. A summary of the characteristics of each of the six classes in the final employment typology can be found in **table S2**, and the conditional item-probabilities can be found in **table S3.** A seventh type, constituted by the unemployed, was added separately.

Second step

Employment trajectories were created by grouping the combinations of unemployment and employment types (hereafter known as employment states) across the five years. Grouping was governed by adhering to patterns guided by the hypotheses of the study: constancy, fluctuation and directional mobility (upwards, downwards, within, between). Initially, there were 8230 potential combinations/trajectories. As based on a previous study where similar employment types emerged (Jonsson et al 2021), unemployment, precarious employment relationship and solo self-employment were grouped as low quality, while standard employment relationship with high income, standard employment relationship, and *business ownership* were grouped as high quality. Hybrid multiple job-holding could not be determined as either high or low quality in this study and was hence kept separate. First, 68 employment trajectories were created by adhering to the patterns. However, in order to make the number of trajectories feasible for further analysis, these trajectories were merged in order to reduce the number. The final number of trajectories reached was 21. See **Table 1** and **Table S4** for details on how specific combinations of employment states were included under these patterns and merged to the final 21 trajectories.

The rationale for manually creating trajectories, versus applying methods such as sequence analysis where trajectories result directly from the analysis, was the ability to categorize individuals in patterns considered relevant the hypotheses.

References

Kreshpaj B. Orellana C. Burström B. Davis L. Hemmingsson T. Johansson G. et al. What is precarious employment? A systematic review of definitions and operationalizations from quantitative and qualitative studies. Scandinavian journal of work. environment & health. 2020;46(3):235-47. doi: 10.5271/sjweh.3875.

Jonsson J, Matilla-Santander N, Kreshpaj B, Orellana C, Johansson G, Burström B, et al. Exploring multidimensional operationalizations of precarious employment in Swedish register data - a typological approach and a summative score approach. Scandinavian journal of work, environment & health. 2021;47(2):117–26.

Nylund-Gibson K. Choi A. Ten frequently asked questions about latent class analysis. 2018;4:440-61. doi: 10.1037/tps0000176.

Collins LM. Lanza ST. Latent Class and Latent Transition Analysis: With Applications in the Social. Behavioral. and Health Sciences. Hoboken: Hoboken: John Wiley & Sons. Incorporated; 2009

Table S1. Complete and exploratory fit indices for the total population, women and men.

Complete and exploratory fit indices				
	**AIC**	**BIC**	**SABIC**	**Entropy**
**Total population**				
**Complete**				
6 classes	94301864.171	94303064.692	94302800.913	0.681
**Exploratory**				
5 classes	50657839.889	50658593.944	50658419.151	0.827
6 classes	47155243.118	47156386.101	47156122.322	0.680
7 classes	47145420.728	47146756.502	47146448.231	0.694
**Women**				
**Complete**				
6 classes	39082202.677	39083340.021	39083076.243	0.690
**Exploratory**				
4 classes	19601268.779	19601984.319	19601809.526	0.819
5 classes	19569212.509	19570110.186	19569890.900	0.811
6 classes	19546346.763	19547426.578	19547162.799	0.692
**Men**				
**Complete**				
6 classes	52698334.651	52699482.926	52699219.148	0.670
**Exploratory**				
4 classes	26446192.612	26446915.396	26446740.604	0.763
5 classes	26398356.226	26399262.992	26399043.707	0.605
6 classes	26356054.044	26357144.792	26356881.014	0.668
AIC= Akaike information criterion; BIC= Bayesian information criterion; SABIC= Sample-size adjusted Bayesian information criterion				

Table S2. Characteristics of the final employment typology with six classes

Class	Characteristics
**Precarious Employment Relationship**	Directly employed or employed by an agency with unstable employment and one job. Mainly median or around median income, high to moderate collective bargaining agreement coverage.
**Solo Self-employment**	Solo self-employed with stable employment and one job. Poor income and poor collective bargaining agreement coverage.
**Hybrid Multiple Job-Holding**	In combined employment (directly employed and self-employed). Unstable employment and the largest proportions of multiple jobs and multiple jobs in multiple sectors. Income and collective bargaining agreement coverage distributed across all levels.
**Business Ownership**	Self-employed with stable employment and one job. Median to high income and poor to moderate collective bargaining agreement coverage.
**Standard Employment Relationship**	Directly employed with stable employment and one job. Median or right below median income with high collective bargaining agreement coverage.
**Standard Employment Relationship with high income**	Directly employed with stable employment and one job. High income and high collective bargaining agreement coverage.

Table S3. Distribution of conditional item-probabilities for the six-class solution (average across 2005-2009) for the total population, women and men.																		
	**Total population N= 2,743,764**						**Women N= 1,275,850**						**Men N= 1,467,914**					
	**PER**	**SSE**	**HMJH**	**BO**	**SER**	**SER/HI**	**PER**	**SSE**	**HMJH**	**BO**	**SER**	**SER/HI**	**PER**	**SSE**	**HMJH**	**BO**	**SER**	**SER/HI**
**Total**	12.9	4.0	4.1	2.5	49.3	27.3	9.8	2.8	4.3	1.2	60.5	21.3	8.9	7.0	5.9	5.9	31.7	40.6
**Contractual relationship insecurity**																		
Directly employed by the employer	98.0	0.0	4.9	0.0	92.4	87.4	98.0	15.4	5.6	0.0	93.3	90.7	98.1	3.9	4.3	16.6	91.3	86.6
Employed by an agency	2.0	0.0	0.0	0.0	0.3	0.0	2.0	0.0	0.0	0.0	0.3	0.2	1.9	0.0	0.0	0.0	0.3	0.1
Combination of self-employed and employment	0.0	9.8	95.0	0.0	7.3	11.8	0.0	7.8	94.4	0.0	6.5	8.4	0.0	16.4	95.5	0.0	8.4	12.4
Self-employed with employees	0.0	12.3	0.1	100.0	0.0	0.8	0.0	8.3	0.0	100.0	0.0	0.7	0.0	10.2	0.2	83.4	0.0	0.9
Solo self-employed	0.0	77.9	0.0	0.0	0.0	0.0	0.0	68.6	0.0	0.0	0.0	0.0	0.0	69.5	0.0	0.0	0.0	0.0
**Contractual temporariness**																		
Stable employment	0.0	87.0	36.0	88.6	100.0	85.5	0.3	89.2	39.3	89.6	100.0	85.9	0.0	88.3	33.8	90.5	100.0	85.3
Unstable employment	100.0	13.0	64.0	11.4	0.0	14.5	99.7	10.9	60.7	10.4	0.0	14.1	100.0	11.7	66.3	9.5	0.0	14.7
**Multiple jobs/economic sectors**																		
1 or 2 employers	80.4	99.8	58.6	99.8	99.2	93.4	80.4	99.9	54.2	99.7	99.6	90.8	80.3	99.8	55.0	99.8	97.7	96.1
≥2 employers	14.9	0.2	29.3	0.2	0.3	5.1	15.0	0.1	32.3	0.3	0.3	6.5	15.0	0.2	31.5	0.2	1.5	2.6
≥2 employers in >1 economic sector	4.7	0.0	12.2	0.0	0.6	1.6	4.6	0.0	13.6	0.0	0.2	2.7	4.7	0.0	13.5	0.0	0.8	1.4
**Income level**																		
≥200% of the median	3.2	2.2	6.7	7.6	0.2	17.6	1.5	2.2	5.6	4.5	0.0	12.1	4.8	2.2	11.8	9.7	0.0	19.6
120-199% of the median	13.6	12.9	22.5	40.6	0.9	80.0	8.2	8.7	18.8	30.2	0.3	82.3	18.3	12.4	27.6	48.6	0.0	80.1
80-119% of the median	60.3	22.5	44.4	45.1	72.0	2.4	56.4	14.3	37.8	42.3	63.2	5.6	63.4	31.1	36.4	37.2	89.0	0.2
60-79% of the median	15.0	22.4	10.5	3.8	19.4	0.0	22.5	11.0	17.2	17.9	27.0	0.0	8.6	19.6	8.4	2.7	8.2	0.0
<60% of the median	7.9	40.1	16.0	2.9	7.5	0.0	11.4	63.8	20.6	5.0	9.5	0.0	4.9	34.7	15.8	1.9	2.7	0.1
**Collective bargaining agreement coverage**																		
>90%	58.3	0.0	28.0	0.4	80.6	86.9	68.7	0.0	40.6	1.1	88.0	90.5	49.3	0.0	30.0	0.1	72.0	85.6
71-90%	30.4	0.0	22.0	49.0	13.8	13.1	23.4	0.0	18.1	43.7	9.4	9.6	36.4	0.0	24.5	40.9	21.3	14.4
≤70%	11.4	100.0	49.9	50.6	5.6	0.0	7.9	100.0	41.3	55.2	2.6	0.0	14.3	100.0	45.5	59.0	6.7	0.0

PER=precarious employment relationship; SSE= solo self-employment; HMJH=hybrid multiple job-holding; BO= business ownership; SER= standard employment relationship; SER/HI= standard employment relationship - high income

Table S4. Grouping of 68 trajectories into the final 21 trajectories

68 trajectories	21 trajectories
**Constant trajectories**	
Classification: Spending 4-5 years in a specific employment state, or in combinations of LQ (UE, PER, SSE) or HQ (BO, SER, SER/HI) employment states, independent of order through time.	
Constant UE	Constant UE
Constant PER	Constant PER
Constant SSE	Constant SSE
Constant HMJH	Constant HMJH
Constant BO	Constant BO
Constant SER	Constant SER
Constant SER/HI	Constant SER/HI
Constant LQ	Constant LQ
Constant HQ	Constant HQ
Abbreviations: UE=unemployment; PER=precarious employment relationship; SSE=solo self-employment; HMJH=hybrid multiple job-holding; BO= business ownership; SER= standard employment relationship; SER/HI=standard employment relationship with high income; LQ=low quality; HQ=high quality.	
**Fluctuating trajectories**	
Classification: Spending a minimum of two years in two different employment states with fluctuating movement in-and-out of the these. Combinations of LQ- and HQ-employment states are also included (if a minimum of two years in the LQ- and HQ-classes, respectively).	
FL PER and UE	FL LQ
FL PER and SSE	
FL UE and SSE	
FL SSE and HMJH	FL LQ and HMJH
FL PER and HMJH	
FL UE and HMJH	
FL HMJH and LQ	
FL PER and SER	FL LQ and HQ
FL PER and SER/HI	
FL SSE and SER	
FL SSE and SER/HI	
FL SSE and BO	
FL LQ and HQ	
FL UE and SER/HI	
FL UE and SER	
FL SER and HMJH	FL HQ and HMJH
FL SER/HI and HMJH	
FL HMJH and BO	
FL HMJH and HQ	
FL SER and SER/HI	FL HQ
FL BO and SER	
FL BO and SER/HI	
Abbreviations: FL=fluctuating; UE=unemployment; PER=precarious employment relationship; SSE=solo self-employment; HMJH=hybrid multiple job-holding; BO= business ownership; SER= standard employment relationship; SER/HI=standard employment relationship with high income; LQ=low quality; HQ=high quality.	
**Trajectories characterized by mobility**	
Classification: Spending a minimum of one year in one employment state at the beginning of the trajectory, followed by a minimum of two years in another employment state at the end of the trajectory. The last two years could also be characterized by combinations of LQ- or HQ-employment states. Mobility can be upwards, downwards, within or between.	
BW PER and UE	WI LQ
BW SSE and UE	
BW SSE and PER	
BW PER and SSE	
BW UE and PER	

BW UE and SSE	
BW SER and SER/HI	WI HQ
BW SER and BO	
BW BO and SER/HI	
BW SER/HI and SER	
BW BO and SER	
BW SER/HI and BO	
BW LQ and HMJH	BW LQ & HMJH
BW HMJH and LQ	
BW HQ and HMJH	BW HQ & HMJH
BW HMJH and HQ	
DW BO to UE	DW HQ to LQ
DW BO to PER	
DW SER to PER	
DW SER/HI to PER	
DW BO to PER-SE	
DW SER/HI to SSE	
DW SER to SSE	
DW SER to UE	
DW SER/HI to UE	
DW HQ to LQ	
UW PER to SER	UW LQ to HQ
UW PER to SER/HI	
UW PER to BO	
UW SSE to BO	
UW SSE to SER/HI	
UW SSE to SER	
UW UE to SER	
UW UE to SER/HI	
UW UE to BO	
UW LQ to HQ	
Abbreviations: BW=between; WI=within; DW= downward; UW=upward; UE=unemployment; PER=precarious employment relationship; SSE=solo self-employment; HMJH=hybrid multiple job-holding; BO= business ownership; SER= standard employment relationship; SER/HI=standard employment relationship with high income; LQ=low quality; HQ=high quality.	
**Other trajectories**	
Any other combination of employment states.	
Other	Other
Note: if potential combinations of employment states matched several patterns, grouping was done in the following hierarchy: fluctuation, constancy, mobility.	

Table S5. Sociodemographic characteristics of the total population (2005) according to employment trajectories (n= 2,743,764).

	Constant									Fluctuating					Mobility						Other	Total
	SER	SER /HI	BO	HMJH	SSE	PER	UE	HQ	LQ	HQ	LQ	HQ and HMJH	LQ and HMJH	LQ and HQ	WI HQ	BW HQ and HMJH	BW LQ and HMJH	WI LQ	UW LQ to HQ	DW HQ to LQ	Other	
	%	%	%	%	%	%	%	%	%	%	%	%	%	%	%	%	%	%	%	%	%	%
**Total**	38.6	21.8	1.7	1.6	2.9	3.8	0.05	4.9	0.6	2.4	0.07	1.1	0.50	7.9	0.2	1.4	0.57	0.02	4.6	4.2	1.46	100
**Age (2005)**																						
18 to 24y	3.1	0.5	0.5	2.9	0.9	20.1	3.6	2.9	9.1	2.4	8.8	7.5	7.4	8.2	3.0	7.3	7.1	8.5	10.2	7.2	7.2	4.1
25 to 34y	19.0	15.7	13.1	17.5	11.0	36.0	22.8	26.3	29.6	24.4	28.3	29.7	24.6	30.9	24.8	24.5	24.9	27.0	32.6	29.3	27.7	21.4
35 to 44y	28.5	33.3	33.8	29.1	27.7	24.5	27.7	32.4	31.4	31.6	30.4	30.9	29.9	30.7	32.4	29.9	30.0	29.6	28.0	31.4	32.3	30.2
45 to 54y	30.6	32.3	33.8	31.8	34.5	14.3	26.4	24.3	20.8	27.2	23.6	21.9	24.7	20.7	26.0	24.6	24.7	23.7	20.2	21.5	22.2	28.2
55 to 61y	18.8	18.2	18.8	18.7	25.8	5.2	19.5	14.1	9.1	14.3	8.9	10.1	13.5	9.4	13.8	13.5	13.4	11.3	8.9	10.5	10.6	16.1
**Level of education (2005)**																						
Primary/secondary school	71.8	39.3	75.4	60.9	78.5	74.3	75.8	58.2	75.7	60.6	77.4	69.0	67.1	70.3	68.2	59.9	66.3	75.6	68.1	70.3	64.7	63.2
Tertiary education <3y	13.4	21.3	12.7	16.6	10.8	11.8	11.2	16.0	11.6	15.6	10.5	12.9	15.0	12.5	13.6	15.5	15.6	10.5	12.2	13.2	15.2	15.2
Tertiary education ≥3y	14.8	39.4	12.0	22.6	10.7	13.9	13.0	25.8	12.7	23.8	12.1	18.0	17.9	17.1	18.2	24.8	18.1	13.9	19.7	16.4	20.1	21.6
**Marital status (2005)**																						
Couple with children	48.1	53.7	59.9	53.0	49.9	41.0	33.7	50.4	44.5	50.0	37.3	48.1	49.6	47.1	47.4	55.0	49.6	41.2	45.2	47.8	51.7	49.5
Couple with no children	16.1	14.9	16.9	14.7	18.0	6.7	12.1	12.2	8.6	12.7	8.6	10.2	11.2	9.8	13.3	12.9	10.8	10.3	9.7	10.4	10.3	13.9
Single with children	9.4	5.9	4.7	7.2	7.3	9.3	13.6	7.2	9.5	7.2	11.4	8.1	7.8	8.3	7.0	6.6	7.9	7.1	8.5	8.7	7.4	8.0
Single	26.4	25.5	18.5	25.1	24.8	43.0	40.5	30.2	37.4	30.1	42.7	33.7	31.4	34.8	32.3	25.5	31.6	41.4	36.6	33.1	30.6	28.5
**Parental mental disorder diagnosis**																						
No	81.4	84.1	85.3	85.7	79.3	84.2	65.5	83.6	77.7	83.1	74.7	83.3	83.3	82.9	83.9	86.2	83.6	75.0	83.4	82.3	83.8	82.7
Yes	11.5	11.0	11.0	10.9	12.0	11.2	13.4	10.7	12.0	10.9	12.2	10.7	10.9	10.7	10.7	10.5	10.8	11.8	10.8	11.3	10.8	11.2
Unknown	7.1	4.9	3.7	3.5	8.7	4.6	21.0	5.7	10.3	5.9	13.1	6.0	5.8	6.3	5.4	3.3	5.6	13.2	5.8	6.5	5.4	6.1
**Parental socioeconomic position during childhood**																						
Manual	51.1	37.9	43.9	35.2	39.1	45.3	9.5	43.1	34.5	45.7	21.8	44.6	32.8	45.5	46.5	38.5	34.8	33.7	43.8	45.4	37.9	44.9
Non-manual	26.9	45.8	35.1	35.3	28.0	33.3	5.3	36.8	23.0	34.3	13.5	33.4	34.6	33.1	33.9	35.8	35.3	26.8	32.3	33.3	37.2	33.7

Farmers. self-employed	8.8	6.7	12.7	20.2	18.5	7.9	1.4	8.4	6.1	8.3	3.1	8.7	15.2	7.6	8.1	16.8	15.3	6.4	7.6	7.9	11.2	8.9
Unknown	13.2	9.7	8.4	9.3	14.4	13.5	83.8	11.7	36.4	11.8	61.6	13.2	17.3	13.8	11.4	8.9	14.6	33.0	16.3	13.4	13.7	12.6

Note: PER=precarious employment relationship; SSE= solo self-employment; HMJH=hybrid multiple job-holding; BO= business ownership; SER= standard employment relationship; SER/HI= standard employment relationship with high income; LQ=low quality; HQ=high quality; WI=within; BW=between; UW=upwards; DW=downwards

Table S6a. Sociodemographic characteristics of women according to employment trajectories (n= 1,275,850)

	Constant									Fluctuating					Mobility						Other	Total
	SER	SER /HI	BO	HMJH	SSE	PER	UE	HQ	LQ	HQ	LQ	HQ and HMJH	LQ and HMJH	LQ and HQ	WI HQ	BW HQ and HMJH	BW LQ and HMJH	WI LQ	UW LQ to HQ	DW HQ to LQ	Other	
	%	%	%	%	%	%	%	%	%	%	%	%	%		%	%	%	%	%	%		%
**Total**	52.5	12.9	0.68	1.46	1.75	3.83	0.07	4	0.6	1.83	0.06	0.86	0.36	7.46	0.15	1.21	0.44	0.01	4.77	4.02	1.01	100
**Age (2005)**																						
18 to 24y	2.3	0.1	0.4	2.2	1.3	21.2	6.2	1.3	10.8	0.9	7.0	7.1	9.5	7.8	2.4	1.5	7.5	6.3	9.3	7.1	8.3	3.7
25 to 34y	17.7	12.5	10.9	14.6	11.5	36.0	22.7	24.0	27.2	25.3	29.3	29.2	23.0	30.6	23.3	16.6	23.5	24.1	32.3	28.9	25.3	20.2
35 to 44y	28.9	32.1	31.9	30.2	28.0	24.5	31.4	33.9	31.6	29.6	31.0	31.4	31.1	31.3	31.3	32.5	32.3	35.1	28.0	31.9	33.5	29.8
45 to 54y	31.9	35.3	36.8	34.9	33.4	13.6	23.6	26.1	19.8	28.5	22.9	22.3	24.9	21	28.2	33.8	24.7	21.3	21.4	21.9	23.2	29.5
55 to 61y	19.3	19.9	20.0	18.1	25.8	4.8	16.1	14.7	10.6	15.7	9.8	9.9	11.6	9.3	14.9	15.6	12.0	13.2	9.0	10.2	9.7	16.7
**Country of birth**																						
Sweden	89.8	90.6	91.2	92.9	87.5	90.8	75.8	89.9	86	90.1	82.5	93.2	88.9	88.9	89.7	92.8	90.9	79.2	89.6	88.7	91.0	89.9
Within EU-28	7.0	6.9	6.6	5.0	8.3	5.2	11.9	6.9	8.0	6.7	9.3	4.7	6.9	6.9	6.9	5.0	5.7	8.9	6.6	6.9	5.6	6.8
Outside EU-28	3.2	2.4	2.2	2.1	4.1	4.0	12.4	3.2	5.9	3.2	8.1	2.1	4.1	4.1	3.4	2.2	3.3	11.9	3.9	4.4	3.4	3.3
**Level of education (2005)**																						
Primary/secondary school	66.1	24.9	68.8	52.0	73.7	66.6	69.4	39.9	73.1	37.9	70.4	62.4	61.4	63.9	53.1	49.4	57.9	74.1	61.2	63.7	57.3	58.2
Tertiary education <3y	15.6	21.8	14.9	18.6	12.8	14.1	14.0	19.4	12.6	18.7	12.8	14.5	16.6	14.2	17.8	17.2	18.3	9.8	13.7	15.5	17.5	16.4
Tertiary education ≥3y	18.4	53.3	16.3	29.4	13.5	19.2	16.5	40.7	14.3	43.3	16.7	23.0	22.1	21.8	29.1	33.4	23.8	16.1	25.2	20.8	25.2	25.4
**Marital status (2005)**																						
Couple with children	50.0	47.3	55.2	51.5	45.1	40.5	40.5	49.8	44.6	47.3	38.4	48.1	46.6	47.3	41.3	55.2	46.7	36.2	44.8	47.9	50.1	48.6
Couple with no children	17.8	16.4	22.4	16.0	22.1	7.1	15.1	13.0	10.6	15.0	10.7	11.2	11.3	10.8	15.3	14.4	11.9	14.9	10.6	11.1	11.2	15.7
Single with children	11.5	11.8	8.2	11.0	11.0	12.8	15.9	12.0	14.7	12.2	19.1	12.2	12.6	12.4	12.8	10.2	12.9	14.9	12.2	13.4	12.4	11.8
Single	20.7	24.5	14.2	21.5	21.7	39.6	28.5	25.2	30.1	25.5	31.8	28.5	29.4	29.5	30.7	20.1	28.5	33.9	32.5	27.7	26.4	23.9
**Age (2009)**																						

22 to 34y	10.7	4.7	4.3	9.0	6.1	44.3	18.5	12.3	26.6	11.7	21.4	23.3	21.4	25	14.6	8.7	19.9	19.0	28.4	22.5	21.4	13.8
35 to 44y	26.1	25.9	24.9	24.3	21.7	28.9	29.8	34.2	30.8	32.9	33.2	32.9	29.6	33.4	29.3	28.0	30.5	31.0	31.0	33.6	32.7	27.8
45 to 54y	31.0	35.2	35.8	35.5	32.5	17.5	27.2	29.3	25.1	28.5	27.2	26.0	29.4	24.9	29.9	35.1	28.7	28.2	23.7	25.9	28.4	30.0
55 to 65y	32.2	34.2	35.0	31.1	39.7	9.3	24.5	24.2	17.4	26.9	18.2	17.9	19.6	16.7	26.2	28.2	20.9	21.8	16.9	18.1	17.4	28.4
**Level of education (2009)**																						
Primary/secondary school	65.2	24.6	68.6	50.1	73.2	61.8	67.4	39.1	70.9	37.3	68.9	60.5	57.5	62	52.4	48.0	55.0	73.6	59.6	61.1	54.0	57.0
Tertiary education <3y	15.5	21.5	14.9	18.6	12.9	14.0	15.0	18.9	13.4	18.4	13.0	13.9	17.8	13.5	17.4	17.0	18.6	9.8	13.5	15.0	18.1	16.3
Tertiary education ≥3y	19.3	53.8	16.5	31.2	13.9	24.2	17.6	42.0	15.7	44.2	18.2	25.6	24.7	24.5	30.2	35.0	26.4	16.7	26.9	24.0	27.8	26.7
**Marital status (2009)**																						
Couple with children	45.8	43.1	48.6	46.3	39.7	44.9	37.5	49.2	43.7	50.5	38.6	49.3	45.4	49.2	45.1	49.9	45.4	33.9	48.8	48.1	48.8	46.1
Couple with no children	23.4	22.3	29.3	22.3	27.9	9.8	17.5	17.1	14.4	17.8	13.1	14.7	15.3	14	19.7	20.8	16.5	16.1	14.1	14.5	15.0	20.7
Single with children	10.7	11.0	8.1	10.6	10.1	12.2	16.4	12.0	14.6	11.1	18.2	11.8	13.1	12	11.8	10.1	12.5	16.7	11.7	12.5	12.4	11.1
Single	20.1	23.6	14.0	20.9	22.3	33.2	28.6	21.7	27.3	20.6	30.1	24.1	26.2	24.7	23.4	19.2	25.6	33.3	25.3	24.8	23.8	22.1
**Mental disorder diagnosis before 2003**																						
No	96.9	97.9	97.6	97.2	96.9	96.6	95.6	97.5	95.8	97.1	94.6	96.9	96.6	96.8	97.3	97.7	96.9	95.4	96.8	96.8	96.8	97.0
Yes	3.1	2.1	2.4	2.8	3.1	3.4	4.4	2.5	4.2	2.9	5.4	3.1	3.4	3.2	2.7	2.3	3.1	4.6	3.2	3.2	3.2	3.0
**Parental mental disorder diagnosis**																						
No	81.6	82.6	83.1	84.6	79.0	84.6	71.4	82.8	79.2	82.7	76.4	83.0	82.9	82.7	82.9	85.5	83.4	74.7	82.9	81.6	83.9	82.2
Yes	11.4	11.0	11.0	10.9	12.3	10.8	11.1	10.8	12.3	10.9	12.1	10.6	11.5	10.6	10.8	10.5	11.2	12.1	10.8	11.3	10.9	11.2
Unknown	6.9	6.5	5.9	4.5	8.8	4.6	17.4	6.4	8.6	6.4	11.5	6.4	5.6	6.6	6.3	4.1	5.3	13.2	6.2	7.1	5.3	6.6
**Parental socioeconomic position during childhood**																						
Manual	50.2	33.0	40.4	34.8	41.8	42.9	23.6	37.6	36.4	38.4	28.2	43.2	33.4	44	42.2	36.5	34.7	24.1	42.5	43.7	37.5	44.8
Non-manual	28.1	49.1	38.0	39.3	33.0	35.3	18.8	42.1	25.3	41.4	18.8	34.7	38.5	34.3	37.8	38.1	39.7	27.0	33.7	34.7	38.8	33.6

Farmers. self-employed	9.0	6.7	10.7	15.4	10.1	8.5	5.3	8.2	7.2	8.2	5.4	8.7	10.8	7.9	7.6	15.9	10.8	5.7	7.8	7.9	10.4	8.7
Unknown	12.7	11.2	10.9	10.5	15.1	13.2	52.3	12.1	31.2	12.0	47.6	13.4	17.4	13.8	12.4	9.5	14.8	43.1	16.0	13.6	13.3	12.9

Note: PER=precarious employment relationship; SSE= solo self-employment; HMJH=hybrid multiple job-holding; BO= business ownership; SER= standard employment relationship; SER/HI= standard employment relationship with high income; LQ=low quality; HQ=high quality; WI=within; BW=between; UW=upwards; DW=downward

Table S6b. Sociodemographic characteristics of men according to trajectories (n=1,467,914)

	Constant									Fluctuating					Mobility						Other	Total
	SER	SER/HI	BO	HMJH	SSE	PER	UE	HQ	LQ	HQ	LQ	HQ and HMJH	LQ and HMJH	LQ and HQ	WI HQ	BW HQ and HMJH	BW LQ and HMJH	WI LQ	UW LQ to HQ	DW HQ to LQ	Other	
	%	%	%	%	%	%	%	%	%	%	%	%	%		%	%	%	%	%	%	%	%
**Total**	25.07	28.97	2.91	1.66	4.45	3.75	0.17	5.59	0.89	2.74	0.11	1.26	0.66	8.49	0.19	1.54	0.70	0.03	4.44	4.53	1.86	
**Age (2005)**																						
18 to 24y	4.6	0.6	0.5	3.4	0.9	19.2	3.9	4.0	10.1	3.3	11.0	2.0	6.9	8.4	3.8	3.4	6.8	9.3	11.1	7.1	6.4	4.4
25 to 34y	21.6	17.1	13.7	17.4	11.1	36.0	22.5	27.9	29.3	23.9	27.1	21.9	25.3	31	26.0	22.8	25.2	30.1	33.1	29.3	28.8	22.4
35 to 44y	27.9	33.8	34.0	26.3	27.5	24.4	30.6	31.4	29.8	32.8	29.4	32.2	29.9	30.2	31.9	32.1	28.7	26.6	28.1	30.9	32.0	30.5
45 to 54y	28.2	31.0	33.1	31.2	34.7	14.9	27.1	23.1	21.4	26.5	23.2	29.0	24.3	20.6	25.1	27.4	24.6	23.8	19.0	21.4	21.9	27.1
55 to 61y	17.7	17.4	18.7	21.7	25.8	5.6	16.0	13.6	9.4	13.6	9.3	14.9	13.6	9.8	13.2	14.3	14.7	10.3	8.7	11.2	11.0	15.6
**Country of birth**																						
Sweden	89.1	93.2	93.9	94.4	88.4	90.9	81.2	91.4	82.6	90.9	77.4	94.6	89.9	89.9	91.3	93.7	91.2	83.1	90.7	90	91.2	91.0
Within EU-28	7.0	4.7	4.5	3.5	6.8	4.7	8.4	5.6	8.6	6.2	11.5	3.4	6.1	5.4	6.1	3.9	4.9	7.8	5.6	6.2	4.9	5.7
Outside EU-28	3.9	2.1	1.5	2.1	4.8	4.3	10.4	3.0	8.8	2.9	11	1.9	3.9	4.6	2.6	2.4	3.9	9.1	3.7	3.8	3.9	3.4
**Level of education (2005)**																						
Primary/secondary school	82.2	45.2	74.8	57.3	80.7	81.0	72.4	70.0	79.5	73.5	82.4	58.3	70.2	75.6	80.2	62.1	70.1	77.1	74.5	75.7	68.5	67.6
Tertiary education <3y	9.3	21.2	13.0	15.7	10.0	9.8	12.4	13.8	10.5	14.0	9.3	15.0	14.3	11.1	10.2	15.2	13.8	10.1	11.0	11.4	13.7	14.1
Tertiary education ≥3y	8.4	33.5	12.2	27.0	9.3	9.2	15.2	16.1	9.9	12.5	8.3	26.7	15.6	13.3	9.6	22.7	16.1	12.8	14.5	12.9	17.8	18.4
**Marital status (2005)**																						
Couple with children	44.9	56.1	60.6	52.7	51.8	41.4	44.6	50.7	45.5	51.7	39.2	55.3	51.4	47.1	49.7	54.6	50.4	42.9	45.9	47.7	52.8	50.3
Couple with no children	12.8	14.3	15.8	14.8	16.4	6.4	10.6	11.7	8.3	11.5	8.3	12.3	10.3	9.2	11.7	11.8	10.7	8.3	8.7	10.1	9.9	12.4

Single with children	5.5	3.7	4.0	4.9	5.9	6.1	7.7	4.4	6.3	4.5	7.0	4.7	5.7	5	4.3	4.9	5.6	4.5	5.1	5.1	5.0	4.8
Single	36.8	25.9	19.6	27.6	25.9	46.1	37.1	33.3	39.9	32.3	45.5	27.7	32.5	38.7	34.3	28.7	33.3	44.4	40.3	37.2	32.3	32.5
**Age (2009)**																						
22 to 34y	15.9	7.2	5.1	12.1	5.5	42.0	15.2	18.7	26.9	15.1	26.0	12.2	20.2	25.6	16.4	14.4	20.1	27.6	30.5	22.9	21.3	15.7
35 to 44y	27.0	30.8	28.9	23.8	21.4	29.0	29.8	33.0	31.0	32.6	29.6	31.2	30.3	33.2	33.5	31.5	29.6	29.1	31.7	33.4	33.9	29.8
45 to 54y	27.9	32.3	34.6	29.0	32.9	18.5	28.6	26.0	25.3	28.7	27.0	30.8	26.7	24.1	27.2	29.4	26.7	25.3	22.2	24.8	26.0	28.4
55 to 65y	29.1	29.6	31.4	35.2	40.3	10.5	26.5	22.3	16.8	23.6	17.4	25.9	22.8	17.1	22.9	24.7	23.7	18.0	15.6	18.9	18.8	26.1
**Level of education (2009)**																						
Primary/secondary school	81.7	45.1	74.7	56.0	80.5	78.7	71.6	69.4	78.5	73.1	81.6	57.6	68.7	74.5	79.3	60.9	68.4	76.9	73.5	74.4	67.3	66.9
Tertiary education <3y	9.5	21.2	13.0	15.5	10.0	10.2	12.6	13.8	10.9	13.9	9.4	15.2	14.5	11.3	10.4	15.4	14.3	11.1	11.2	11.7	14.0	14.2
Tertiary education ≥3y	8.8	33.7	12.3	28.5	9.4	11.0	15.8	16.8	10.6	12.9	9.0	27.2	16.8	14.2	10.2	23.7	17.3	12.1	15.3	13.9	18.7	18.9
**Marital status (2009)**																						
Couple with children	43.1	54.0	56.1	49.1	46.3	44.1	42.0	51.9	45.6	51.7	40.4	54.7	50.4	49.5	49.1	54.0	49.6	44.4	49.0	49.0	53.8	49.4
Couple with no children	17.2	19.7	22.1	20.9	22.5	9.2	14.1	15.7	11.2	15.6	9.6	16.9	14.0	12.3	16.4	16.3	15.1	11.5	11.7	13.4	13.4	16.9
Single with children	5.3	4.0	4.4	4.7	5.9	5.0	6.9	4.4	6.3	4.8	7.4	5.1	5.4	4.8	4.5	4.8	5.5	5.8	4.6	4.9	5.0	4.8
Single	34.3	22.2	17.4	25.3	25.3	41.7	36.9	27.9	36.9	27.9	42.6	23.3	30.2	33.4	30.0	24.9	29.8	38.3	34.8	32.6	27.9	28.9
**Mental disorder diagnosis before 2003**																						
No	96.5	98.3	98.4	97.9	97.7	96.7	95.7	97.7	96.0	97.5	94.5	98	97.7	97.1	97.2	97.9	97.5	96.2	96.9	97.0	97.5	97.4
Yes	3.5	1.7	1.6	2.1	2.3	3.3	4.3	2.3	4.0	2.5	5.5	2.0	2.3	2.9	2.8	2.1	2.5	3.8	3.1	3.0	2.5	2.6
**Parental mental disorder diagnosis**																						
No	81.0	84.7	85.6	85.3	79.7	83.9	76.4	84.1	77.0	83.4	73.7	90.8	83.8	87.5	84.4	85.7	83.9	74.9	83.9	82.8	84.1	83.2
Yes	11.7	11.0	11.0	11.3	11.8	11.6	12.2	10.7	11.8	11.0	11.5	6.1	10.6	6.4	10.5	10.6	10.5	10.8	10.7	11.2	10.8	11.2

Unknown	7.3	4.3	3.4	3.4	8.5	4.5	11.4	5.2	11.2	5.6	14.7	3.1	5.7	6.1	5.1	3.8	5.6	14.3	5.4	5.9	5.1	5.7
**Parental socioeconomic position during childhood**																						
Manual	53.2	39.9	43.6	33.1	38.4	47.4	23.3	46.5	35.8	49.8	22.2	38.3	33.0	46.7	51.2	38.1	34.8	36.1	45.1	46.8	38.7	45.0
Non-manual	24.5	44.4	35.7	37.2	25.4	31.6	22.5	33.3	22.7	30.4	14.0	37.4	32.6	32.1	28.8	37.1	33.4	26.6	31.1	32.2	36.0	33.7
Farmers. self-employed	8.2	6.6	12.5	20.3	22.2	7.3	6.2	8.6	6.9	8.4	3.2	15.7	17.4	7.6	8.7	15.5	17.3	7.3	7.4	8.0	12.1	9.0
Unknown	14.1	9.1	8.1	9.3	14.1	13.7	48.0	11.5	34.6	11.5	60.7	8.6	17.0	13.6	11.3	9.3	14.6	30.1	16.4	13.0	13.2	12.3

Note: PER=precarious employment relationship; SSE= solo self-employment; HMJH=hybrid multiple job-holding; BO= business ownership; SER= standard employment relationship; SER/HI= standard employment relationship with high income; LQ=low quality; HQ=high quality; WI=within; BW=between; UW=upwards; DW=downward

Table S7a. Hazard ratios (crude and adjusted) for common mental disorders for the total population (n= 2,743,764), women (n= 1,275,850) and men (n=1,467,914).

		Common mental disorders											
Total		No. of cases	HR	CI95%			HR1	CI95%			HR2	CI95%	
	Constant SER	40261	ref	ref	ref		ref	ref	ref		ref	ref	ref
	Constant SER/HI	14450	0.63	0.62	0.64		0.77	0.76	0.79		0.77	0.75	0.78
	Constant BO	1088	0.62	0.58	0.66		0.83	0.78	0.88		0.84	0.79	0.89
	Constant HMJH	1361	0.81	0.77	0.86		0.95	0.90	1.01		0.97	0.92	1.02
	Constant SSE	2362	0.76	0.73	0.79		0.94	0.90	0.98		0.95	0.91	0.99
	Constant PE	5808	1.47	1.43	1.51		1.29	1.25	1.32		1.27	1.24	1.31
	Constant UE	97	1.77	1.45	2.16		1.61	1.31	1.96		1.42	1.16	1.74
	Constant HQ	4439	0.87	0.84	0.89		0.94	0.91	0.96		0.93	0.90	0.96
	Constant LQ	915	1.42	1.33	1.52		1.35	1.27	1.45		1.29	1.21	1.38
	Fluctuating HQ	2079	0.83	0.80	0.87		0.91	0.88	0.96		0.91	0.87	0.95
	Fluctuating LQ	134	1.79	1.51	2.13		1.63	1.38	1.93		1.49	1.26	1.77
	Fluctuating HQ and HMJH	906	0.78	0.73	0.83		0.91	0.85	0.97		0.92	0.86	0.98
	Fluctuating LQ and HMJH	604	1.14	1.05	1.23		1.21	1.12	1.32		1.20	1.11	1.30
	Fluctuating LQ and HQ	9258	1.10	1.08	1.13		1.08	1.05	1.10		1.07	1.05	1.10
	Within HQ	206	0.96	0.84	1.10		1.05	0.92	1.21		1.05	0.92	1.20
	Between HQ and HMJH	1240	0.85	0.80	0.90		0.94	0.89	1.00		0.95	0.90	1.01
	Between LQ and HMJH	653	1.09	1.01	1.18		1.16	1.07	1.25		1.16	1.07	1.25
	Within LQ	37	1.71	1.24	2.37		1.70	1.23	2.35		1.62	1.17	2.23
	Upward LQ to HQ	5438	1.12	1.09	1.15		1.05	1.02	1.08		1.04	1.01	1.07
	Downward HQ to LQ	5030	1.13	1.09	1.16		1.11	1.07	1.14		1.10	1.07	1.13
	Other	1627	1.06	1.01	1.12		1.12	1.07	1.18		1.12	1.06	1.17
Women		No. of cases	HR	CI95%			HR1	CI95%			HR2	CI95%	
	Constant SER	27645	ref	ref	ref		ref	ref	ref		ref	ref	ref

	**Constant SER/HI**	5446	0.80	0.77	0.82		0.84	0.81	0.86		0.83	0.80	0.85
	**Constant BO**	282	0.78	0.70	0.88		0.86	0.76	0.97		0.86	0.76	0.97
	**Constant HMJH**	803	1.05	0.98	1.12		1.08	1.01	1.16		1.08	1.01	1.16
	**Constant SSE**	879	0.95	0.89	1.02		1.00	0.93	1.07		0.99	0.92	1.06
	**Constant PE**	3438	1.73	1.66	1.79		1.32	1.27	1.36		1.30	1.25	1.35
	**Constant UE**	69	1.79	1.42	2.27		1.47	1.16	1.86		1.35	1.06	1.71
	**Constant HQ**	2124	1.01	0.96	1.05		0.96	0.92	1.01		0.96	0.91	1.00
	**Constant LQ**	518	1.66	1.52	1.81		1.36	1.25	1.49		1.30	1.19	1.42
	**Fluctuating HQ**	967	1.00	0.94	1.07		0.96	0.90	1.03		0.95	0.89	1.02
	**Fluctuating LQ**	64	1.92	1.51	2.46		1.50	1.18	1.92		1.38	1.08	1.77
	**Fluctuating HQ and HMJH**	438	0.96	0.88	1.06		1.00	0.91	1.10		1.01	0.92	1.11
	**Fluctuating LQ and HMJH**	264	1.40	1.24	1.59		1.25	1.10	1.41		1.22	1.08	1.38
	**Fluctuating LQ and HQ**	5038	1.29	1.25	1.33		1.11	1.08	1.14		1.10	1.07	1.13
	**Within HQ**	92	1.18	0.96	1.44		1.11	0.91	1.37		1.11	0.90	1.36
	**Between HQ and HMJH**	671	1.05	0.98	1.14		1.06	0.98	1.14		1.06	0.98	1.14
	**Between LQ and HMJH**	334	1.44	1.30	1.61		1.31	1.18	1.46		1.29	1.16	1.44
	**Within LQ**	13	1.85	1.07	3.18		1.52	0.88	2.61		1.40	0.81	2.41
	**Upward LQ to HQ**	3166	1.26	1.22	1.31		1.07	1.03	1.11		1.06	1.02	1.10
	**Downward HQ to LQ**	2796	1.33	1.28	1.38		1.16	1.11	1.20		1.14	1.10	1.19
	**Other**	726	1.37	1.27	1.47		1.21	1.13	1.31		1.20	1.12	1.30
		**No. of cases**	**HR**	**CI95%**			**HR1**	**CI95%**			**HR2**	**CI95%**	
	**Constant SER**	12100	ref	ref	ref		ref	ref	ref		ref	ref	ref
	**Constant SER/HI**	8831	0.63	0.61	0.64		0.68	0.66	0.70		0.68	0.66	0.70
	**Constant BO**	962	0.68	0.64	0.73		0.76	0.71	0.81		0.77	0.72	0.82
**Men**	**Constant HMJH**	642	0.80	0.74	0.86		0.86	0.80	0.94		0.88	0.81	0.95
	**Constant SSE**	1672	0.78	0.74	0.82		0.85	0.81	0.90		0.87	0.82	0.91
	**Constant PE**	2389	1.32	1.27	1.38		1.19	1.14	1.24		1.18	1.13	1.23
	**Constant UE**	121	1.49	1.25	1.78		1.42	1.19	1.70		1.34	1.12	1.61
	**Constant HQ**	2318	0.86	0.82	0.89		0.87	0.83	0.91		0.87	0.83	0.91

	**Constant LQ**	599	1.40	1.29	1.52		1.28	1.18	1.39		1.24	1.14	1.35
	**Fluctuating HQ**	1079	0.81	0.76	0.87		0.83	0.78	0.89		0.83	0.78	0.89
	**Fluctuating LQ**	86	1.72	1.39	2.12		1.46	1.18	1.81		1.37	1.10	1.70
	**Fluctuating HQ and HMJH**	455	0.75	0.68	0.82		0.79	0.72	0.87		0.80	0.73	0.88
	**Fluctuating LQ and HMJH**	335	1.04	0.93	1.16		1.04	0.94	1.16		1.05	0.94	1.17
	**Fluctuating LQ and HQ**	4222	1.02	0.99	1.06		0.98	0.95	1.02		0.98	0.95	1.01
	**Within HQ**	93	1.02	0.83	1.25		1.02	0.83	1.25		1.03	0.84	1.26
	**Between HQ and HMJH**	588	0.78	0.72	0.85		0.82	0.75	0.89		0.83	0.76	0.90
	**Between LQ and HMJH**	337	1.00	0.89	1.11		1.00	0.89	1.11		1.01	0.90	1.12
	**Within LQ**	25	1.94	1.31	2.87		1.79	1.21	2.65		1.74	1.17	2.57
	**Upward LQ to HQ**	2218	1.03	0.99	1.08		0.97	0.93	1.02		0.97	0.92	1.01
	**Downward HQ to LQ**	2237	1.02	0.98	1.07		0.99	0.95	1.04		0.99	0.94	1.03
	**Other**	911	1.01	0.95	1.08		1.00	0.94	1.07		1.01	0.94	1.08

Note 1: HR: not adjusted. HR1: adjusted for sex, age in 2005, level of education in 2005, marital status in 2005, country of birth, any mental disorder diagnosis before 2003. HR2: adjusted for adjusted for sex, age in 2005, level of education in 2005, marital status in 2005, country of birth, any mental disorder diagnosis before 2003, parental mental disorder diagnosis, and parental socioeconomic position during childhood.

Note 2: PER=precarious employment relationship; SSE= solo self-employment; HMJH=hybrid multiple job-holding; BO= business ownership; SER= standard employment relationship; SER/HI= standard employment relationship - high income; LQ=low quality; HQ=high quality

Table S7b. Hazard ratios (crude and adjusted) for substance use disorders for the total population (n= 2,743,764), women (n= 1,275,850) and men (n=1,467,914).

		Substance use disorders											
Total		**No. of cases**	**HR**	**CI95%**			**HR1**	**CI95%**			**HR2**	**CI95%**	
	**Constant SER**	12370	ref	ref	ref		ref	ref	ref		ref	ref	ref
	**Constant SER/HI**	5942	0.84	0.82	0.87		0.81	0.79	0.84		0.80	0.78	0.83
	**Constant BO**	605	1.13	1.04	1.22		0.92	0.85	1.00		0.93	0.86	1.01
	**Constant HMJH**	448	0.88	0.80	0.96		0.81	0.73	0.89		0.84	0.77	0.93
	**Constant SSE**	1133	1.20	1.13	1.27		0.94	0.88	1.00		0.98	0.92	1.04
	**Constant PE**	1705	1.40	1.33	1.47		1.30	1.23	1.37		1.28	1.21	1.34
	**Constant UE**	60	3.56	2.75	4.59		2.45	1.90	3.16		2.19	1.69	2.83
	**Constant HQ**	1549	0.99	0.94	1.04		0.92	0.87	0.97		0.92	0.87	0.97
	**Constant LQ**	394	1.99	1.80	2.20		1.63	1.47	1.80		1.56	1.41	1.72
	**Fluctuating HQ**	829	1.09	1.01	1.17		0.97	0.91	1.04		0.97	0.90	1.04
	**Fluctuating LQ**	65	2.82	2.21	3.60		2.07	1.62	2.64		1.90	1.49	2.43
	**Fluctuating HQ and HMJH**	296	0.84	0.74	0.94		0.77	0.69	0.87		0.79	0.71	0.89
	**Fluctuating LQ and HMJH**	213	1.28	1.12	1.47		1.13	0.99	1.30		1.15	1.00	1.32
	**Fluctuating LQ and HQ**	2948	1.15	1.10	1.19		1.06	1.01	1.10		1.05	1.01	1.09
	**Within HQ**	80	1.22	0.98	1.51		1.05	0.84	1.31		1.05	0.84	1.31
	**Between HQ and HMJH**	357	0.80	0.72	0.89		0.76	0.68	0.85		0.78	0.70	0.87
	**Between LQ and HMJH**	196	1.06	0.92	1.22		0.95	0.82	1.09		0.97	0.84	1.12
	**Within LQ**	14	2.10	1.24	3.54		1.67	0.99	2.82		1.63	0.96	2.75
	**Upward LQ to HQ**	1660	1.11	1.05	1.17		1.06	1.01	1.12		1.05	0.99	1.10
	**Downward HQ to LQ**	1792	1.30	1.24	1.37		1.19	1.14	1.26		1.18	1.12	1.24
	**Other**	501	1.06	0.97	1.16		0.96	0.88	1.05		0.96	0.88	1.05
		**No. of cases**	**HR**	**CI95%**			**HR1**	**CI95%**			**HR2**	**CI95%**	
	**Constant SER**	5245	ref	ref	ref		ref	ref	ref		ref	ref	ref

	**Constant SER/HI**	993	0.77	0.72	0.82		0.95	0.89	1.02		0.94	0.87	1.00
	**Constant BO**	80	1.18	0.94	1.47		1.26	1.01	1.57		1.27	1.01	1.58
	**Constant HMJH**	136	0.93	0.79	1.11		1.02	0.86	1.21		1.04	0.88	1.23
	**Constant SSE**	214	1.23	1.07	1.41		1.19	1.04	1.37		1.19	1.04	1.37
	**Constant PE**	521	1.36	1.24	1.49		1.29	1.17	1.41		1.27	1.16	1.40
	**Constant UE**	8	1.08	0.54	2.17		0.99	0.49	1.97		0.94	0.47	1.89
	**Constant HQ**	377	0.94	0.85	1.05		1.07	0.97	1.19		1.06	0.96	1.18
**Women**	**Constant LQ**	109	1.82	1.50	2.20		1.65	1.37	2.00		1.60	1.32	1.94
	**Fluctuating HQ**	174	0.95	0.82	1.11		1.07	0.92	1.25		1.06	0.91	1.23
	**Fluctuating LQ**	15	2.34	1.41	3.88		1.96	1.18	3.26		1.87	1.13	3.11
	**Fluctuating HQ and HMJH**	75	0.87	0.69	1.09		0.99	0.79	1.25		1.01	0.80	1.27
	**Fluctuating LQ and HMJH**	45	1.21	0.89	1.63		1.20	0.89	1.62		1.19	0.88	1.61
	**Fluctuating LQ and HQ**	806	1.08	1.01	1.16		1.06	0.99	1.15		1.05	0.98	1.14
	**Within HQ**	18	1.21	0.76	1.92		1.26	0.80	2.01		1.26	0.79	2.00
	**Between HQ and HMJH**	94	0.78	0.64	0.96		0.87	0.71	1.06		0.88	0.72	1.08
	**Between LQ and HMJH**	49	1.09	0.82	1.44		1.10	0.83	1.47		1.10	0.83	1.46
	**Within LQ**	2	1.48	0.37	5.93		1.33	0.33	5.30		1.27	0.32	5.08
	**Upward LQ to HQ**	540	1.13	1.03	1.23		1.12	1.03	1.23		1.11	1.01	1.21
	**Downward HQ to LQ**	524	1.30	1.19	1.43		1.28	1.17	1.41		1.27	1.16	1.39
	**Other**	103	1.01	0.83	1.23		1.03	0.85	1.26		1.03	0.84	1.25
		**No. of cases**	**HR**	**CI95%**			**HR1**	**CI95%**			**HR2**	**CI95%**	
	**Constant SER**	6902	ref	ref	ref		ref	ref	ref		ref	ref	ref
	**Constant SER/HI**	4886	0.61	0.59	0.63		0.77	0.74	0.80		0.76	0.73	0.79
	**Constant BO**	620	0.77	0.71	0.83		0.89	0.82	0.97		0.90	0.83	0.98
**Men**	**Constant HMJH**	303	0.66	0.59	0.74		0.78	0.70	0.88		0.81	0.72	0.91
	**Constant SSE**	1013	0.83	0.78	0.88		0.85	0.80	0.91		0.90	0.84	0.96
	**Constant PE**	1189	1.15	1.08	1.22		1.29	1.21	1.37		1.26	1.18	1.34
	**Constant UE**	97	2.10	1.72	2.57		2.15	1.75	2.63		1.99	1.63	2.44
	**Constant HQ**	1176	0.76	0.72	0.81		0.88	0.82	0.93		0.88	0.82	0.93

	**Constant LQ**	362	1.49	1.34	1.65		1.59	1.43	1.77		1.52	1.37	1.70
	**Fluctuating HQ**	638	0.85	0.78	0.92		0.94	0.87	1.02		0.94	0.87	1.02
	**Fluctuating LQ**	52	1.82	1.38	2.38		1.79	1.36	2.35		1.64	1.25	2.16
	**Fluctuating HQ and HMJH**	206	0.59	0.52	0.68		0.71	0.63	0.82		0.73	0.64	0.84
	**Fluctuating LQ and HMJH**	168	0.90	0.77	1.05		1.05	0.90	1.22		1.07	0.91	1.25
	**Fluctuating LQ and HQ**	2155	0.91	0.87	0.96		1.02	0.98	1.08		1.02	0.97	1.07
	**Within HQ**	52	1.00	0.76	1.31		1.08	0.82	1.42		1.09	0.83	1.43
	**Between HQ and HMJH**	266	0.63	0.55	0.71		0.75	0.66	0.85		0.77	0.68	0.87
	**Between LQ and HMJH**	157	0.81	0.69	0.95		0.93	0.79	1.09		0.95	0.81	1.12
	**Within LQ**	12	1.62	0.92	2.85		1.71	0.97	3.02		1.69	0.96	2.98
	**Upward LQ to HQ**	1102	0.90	0.84	0.96		1.01	0.95	1.08		1.00	0.94	1.07
	**Downward HQ to LQ**	1291	1.03	0.97	1.09		1.14	1.07	1.21		1.13	1.07	1.20
	**Other**	382	0.74	0.67	0.82		0.88	0.79	0.98		0.88	0.80	0.98

Note 1: HR: not adjusted. HR1: adjusted for sex, age in 2005, level of education in 2005, marital status in 2005, country of birth, any mental disorder diagnosis before 2003. HR2: adjusted for adjusted for sex, age in 2005, level of education in 2005, marital status in 2005, country of birth, any mental disorder diagnosis before 2003, parental mental disorder diagnosis, and parental socioeconomic position during childhood.

Note 2: PER=precarious employment relationship; SSE= solo self-employment; HMJH=hybrid multiple job-holding; BO= business ownership; SER= standard employment relationship; SER/HI= standard employment relationship - high income; LQ=low quality; HQ=high quality

Table S7c. Hazard ratios (crude and adjusted) for suicide attempt for the total population (n= 2,743,764), women (n= 1,275,850) and men (n=1,467,914).

		Suicide attempt											
Total		**No. of cases**	**HR**	**CI95%**			**HR1**	**CI95%**			**HR2**	**CI95%**	
	**Constant SER**	6350	ref	ref	ref		ref	ref	ref		ref	ref	ref
	**Constant SER/HI**	2864	0.79	0.76	0.83		0.85	0.81	0.90		0.86	0.82	0.90
	**Constant BO**	267	0.97	0.86	1.10		0.94	0.83	1.06		0.95	0.84	1.07
	**Constant HMJH**	255	0.97	0.86	1.10		0.98	0.87	1.12		0.99	0.87	1.12
	**Constant SSE**	474	0.97	0.89	1.07		0.95	0.86	1.04		0.95	0.86	1.04
	**Constant PE**	902	1.44	1.34	1.54		1.22	1.14	1.32		1.22	1.14	1.31
	**Constant UE**	20	2.31	1.49	3.59		1.94	1.25	3.01		1.76	1.13	2.73
	**Constant HQ**	829	1.03	0.96	1.11		1.01	0.94	1.09		1.01	0.94	1.09
	**Constant LQ**	160	1.57	1.34	1.84		1.34	1.15	1.57		1.30	1.11	1.52
	**Fluctuating HQ**	387	0.99	0.89	1.09		0.96	0.87	1.06		0.96	0.87	1.07
	**Fluctuating LQ**	19	1.59	1.01	2.50		1.30	0.83	2.04		1.21	0.77	1.90
	**Fluctuating HQ and HMJH**	198	1.09	0.95	1.26		1.10	0.95	1.26		1.01	0.96	1.27
	**Fluctuating LQ and HMJH**	109	1.31	1.08	1.58		1.22	1.01	1.47		1.21	1.00	1.47
	**Fluctuating LQ and HQ**	1454	1.10	1.04	1.16		1.01	0.95	1.26		1.01	0.95	1.07
	**Within HQ**	35	1.03	0.74	1.44		0.98	0.70	1.36		0.98	0.70	1.37
	**Between HQ and HMJH**	228	0.99	0.87	1.13		0.98	0.86	1.12		0.99	0.87	1.13
	**Between LQ and HMJH**	93	0.97	0.79	1.20		0.90	0.74	1.11		0.90	0.74	1.11
	**Within LQ**	5	1.44	0.60	3.47		1.25	0.52	3.00		1.22	0.51	2.94
	**Upward LQ to HQ**	826	1.08	1.00	1.16		0.99	0.92	1.06		0.98	0.91	1.05
	**Downward HQ to LQ**	833	1.18	1.10	1.27		1.08	1.01	1.17		1.08	1.01	1.17
	**Other**	264	1.09	0.97	1.24		1.01	0.90	1.15		1.01	0.90	1.15
		**No. of cases**	**HR**	**CI95%**			**HR1**	**CI95%**			**HR2**	**CI95%**	
	**Constant SER**	3659	ref	ref	ref		ref	ref	ref		ref	ref	ref
	**Constant SER/HI**	708	0.79	0.72	0.85		0.90	0.82	0.97		0.90	0.82	0.98

**Women**	**Constant BO**	43	0.91	0.67	1.22		0.95	0.70	1.28		0.96	0.71	1.29
	**Constant HMJH**	93	0.92	0.75	1.12		0.96	0.78	1.18		0.97	0.79	1.19
	**Constant SSE**	120	0.98	0.82	1.18		0.99	0.83	1.19		0.99	0.83	1.19
	**Constant PE**	345	1.29	1.16	1.44		1.15	1.03	1.29		1.14	1.02	1.28
	**Constant UE**	5	0.97	0.40	2.33		0.87	0.36	2.08		0.80	0.33	1.92
	**Constant HQ**	263	0.94	0.83	1.07		0.99	0.87	1.12		0.99	0.87	1.12
	**Constant LQ**	50	1.20	0.91	1.59		1.07	0.81	1.42		1.02	0.77	1.35
	**Fluctuating HQ**	128	1.00	0.83	1.19		1.05	0.88	1.25		1.05	0.88	1.25
	**Fluctuating LQ**	6	1.33	0.60	2.97		1.14	0.51	2.54		1.04	0.47	2.33
	**Fluctuating HQ and HMJH**	61	1.02	0.79	1.31		1.08	0.84	1.40		1.09	0.84	1.40
	**Fluctuating LQ and HMJH**	32	1.29	0.91	1.82		1.23	0.87	1.74		1.21	0.85	1.72
	**Fluctuating LQ and HQ**	562	1.01	0.98	1.17		1.02	0.93	1.11		1.02	0.93	1.11
	**Within HQ**	12	1.16	0.66	2.04		1.17	0.67	2.07		1.17	0.67	2.07
	**Between HQ and HMJH**	85	1.01	0.82	1.26		1.06	0.85	1.31		1.06	0.86	1.32
	**Between LQ and HMJH**	31	1.00	0.70	1.43		0.98	0.69	1.39		0.97	0.68	1.38
	**Within LQ**	2	2.12	0.53	8.48		1.84	0.46	7.37		1.73	0.43	6.93
	**Upward LQ to HQ**	326	0.98	0.87	1.10		0.93	0.83	1.04		0.92	0.82	1.03
	**Downward HQ to LQ**	355	1.27	1.14	1.42		1.20	1.07	1.33		1.19	1.07	1.33
	**Other**	72	1.02	0.81	1.29		0.99	0.78	1.25		0.98	0.78	1.24
**Men**		**No. of cases**	**HR**	**CI95%**			**HR1**	**CI95%**			**HR2**	**CI95%**	
	**Constant SER**	2606	ref	ref	ref		ref	ref	ref		ref	ref	ref
	**Constant SER/HI**	2124	0.70	0.66	0.74		0.84	0.79	0.89		0.85	0.80	0.90
	**Constant BO**	257	0.85	0.75	0.96		0.92	0.81	1.05		0.93	0.82	1.06
	**Constant HMJH**	143	0.83	0.70	0.98		0.95	0.80	1.12		0.95	0.81	1.13
	**Constant SSE**	410	0.89	0.80	0.98		0.96	0.87	1.07		0.97	0.87	1.07
	**Constant PE**	562	1.43	1.31	1.57		1.29	1.17	1.41		1.28	1.17	1.41
	**Constant UE**	23	1.32	0.87	1.99		1.34	0.89	2.01		1.30	0.86	1.95
	**Constant HQ**	561	0.97	0.88	1.06		1.00	0.91	1.09		1.00	0.91	1.10
	**Constant LQ**	144	1.56	1.32	1.85		1.47	1.24	1.74		1.45	1.22	1.71

	**Fluctuating HQ**	256	0.90	0.79	1.02		0.93	0.82	1.06		0.93	0.82	1.06
	**Fluctuating LQ**	17	1.56	0.97	2.52		1.43	0.89	2.31		1.37	0.85	2.22
	**Fluctuating HQ and HMJH**	123	0.94	0.78	1.12		1.05	0.87	1.25		1.05	0.87	1.26
	**Fluctuating LQ and HMJH**	81	1.17	0.94	1.47		1.21	0.97	1.51		1.21	0.97	1.51
	**Fluctuating LQ and HQ**	900	1.01	0.78	1.12		0.99	0.92	1.07		0.99	0.92	1.07
	**Within HQ**	18	0.92	0.58	1.45		0.91	0.57	1.45		0.92	0.58	1.46
	**Between HQ and HMJH**	146	0.91	0.77	1.08		0.98	0.83	1.16		0.99	0.83	1.17
	**Between LQ and HMJH**	73	0.99	0.79	1.26		1.01	0.80	1.28		1.01	0.80	1.28
	**Within LQ**	3	1.06	0.34	3.29		1.02	0.33	3.16		1.01	0.33	3.14
	**Upward LQ to HQ**	494	1.07	0.97	1.18		1.03	0.93	1.13		1.02	0.93	1.13
	**Downward HQ to LQ**	487	1.03	0.94	1.14		1.01	0.92	1.12		1.01	0.92	1.12
	**Other**	186	0.96	0.83	1.12		0.98	0.84	1.14		0.98	0.85	1.14

Note 1: HR: not adjusted. HR1: adjusted for sex, age in 2005, level of education in 2005, marital status in 2005, country of birth, any mental disorder diagnosis before 2003. HR2: adjusted for adjusted for sex, age in 2005, level of education in 2005, marital status in 2005, country of birth, any mental disorder diagnosis before 2003, parental mental disorder diagnosis, and parental socioeconomic position during childhood.

Note 2: PER=precarious employment relationship; SSE= solo self-employment; HMJH=hybrid multiple job-holding; BO= business ownership; SER= standard employment relationship; SER/HI= standard employment relationship - high income; LQ=low quality; HQ=high quality

Table S8a. Fully adjusted hazard ratios for specific outcomes (depression, anxiety, stress related disorders, alcohol and non-alcohol use disorders) for the total population (n=2,743,764).

	Depression					Anxiety					Stress related disorders					Alcohol use disorders					Non-alcoholic use disorders			
	cases	HR2	CI95%			cases	HR2	CI95%			cases	HR2	CI95%			cases	HR2	CI95%			cases	HR2	CI95%	
**Constant SER**	21032	ref	ref			17892	ref	ref			15085	ref	ref			10933	ref	ref			2187	ref	ref	
**Constant SER/HI**	7048	0.70	0.68	0.72		5736	0.71	0.69	0.73		5821	0.88	0.85	0.91		5565	0.83	0.80	0.85		628	0.61	0.56	0.67
**Constant BO**	542	0.77	0.71	0.84		460	0.80	0.73	0.88		414	0.96	0.87	1.06		562	0.94	0.86	1.02		69	0.81	0.63	1.03
**Constant HMJH**	669	0.89	0.83	0.96		549	0.89	0.82	0.97		584	1.17	1.07	1.27		397	0.83	0.75	0.91		73	0.91	0.72	1.15
**Constant SSE**	1207	0.89	0.84	0.94		993	0.90	0.85	0.97		851	1.02	0.95	1.09		1048	0.99	0.92	1.05		132	0.87	0.72	1.03
**Constant PER**	2957	1.27	1.23	1.33		2647	1.23	1.18	1.28		2384	1.32	1.27	1.38		1415	1.25	1.18	1.32		450	1.38	1.24	1.53
**Constant UE**	56	1.50	1.15	1.96		46	1.46	1.09	1.96		40	1.62	1.19	2.21		46	1.90	1.42	2.55		22	4.10	2.68	6.26
**Constant HQ**	2232	0.89	0.86	0.94		1866	0.88	0.84	0.93		1818	1.04	0.99	1.09		1405	0.93	0.88	0.99		232	0.81	0.71	0.93
**Constant LQ**	501	1.35	1.23	1.47		407	1.24	1.12	1.37		373	1.40	1.27	1.56		344	1.57	1.41	1.75		91	1.69	1.37	2.10
**Fluctuating HQ**	1039	0.87	0.81	0.92		898	0.89	0.83	0.95		824	1.00	0.93	1.07		742	0.97	0.90	1.04		139	0.99	0.84	1.18
**Fluctuating LQ**	65	1.33	1.04	1.71		55	1.32	1.01	1.72		60	1.77	1.37	2.29		48	1.63	1.22	2.16		25	3.32	2.23	4.93
**Fluctuating HQ and HMJH**	474	0.91	0.83	1.00		363	0.84	0.76	0.94		375	1.07	0.96	1.18		265	0.79	0.70	0.89		48	0.83	0.62	1.11
**Fluctuating LQ and HMJH**	306	1.15	1.03	1.29		261	1.15	1.02	1.30		256	1.38	1.22	1.56		188	1.14	0.98	1.32		39	1.15	0.84	1.59
**Fluctuating LQ and HQ**	4646	1.03	1.01	1.07		3985	1.01	0.97	1.04		3779	1.14	1.10	1.19		2570	1.04	1.00	1.09		608	1.07	0.98	1.18
**Within HQ**	101	0.97	0.80	1.18		94	1.06	0.87	1.30		79	1.13	0.90	1.41		76	1.12	0.89	1.40		10	0.78	0.42	1.46
**Between HMJH and HQ**	612	0.89	0.82	0.97		468	0.81	0.74	0.89		531	1.12	1.03	1.22		329	0.81	0.72	0.90		46	0.61	0.45	0.81
**Between HMJH and LQ**	340	1.14	1.03	1.27		273	1.07	0.95	1.21		265	1.27	1.13	1.44		170	0.95	0.81	1.10		38	1.05	0.76	1.45
**Within LQ**	22	1.79	1.18	2.71		14	1.30	0.77	2.20		17	2.00	1.25	3.23		13	1.73	1.01	2.99		2	1.16	0.29	4.65
**Upward LQ to HQ**	2728	1.01	0.97	1.06		2428	1.01	0.97	1.06		2279	1.13	1.08	1.18		144	1.05	0.99	1.11		331	0.99	0.88	1.11
**Downward HQ to LQ**	2591	1.09	1.04	1.13		2253	1.08	1.03	1.13		2021	1.17	1.11	1.22		1583	1.19	1.13	1.26		346	1.17	1.04	1.31
**Other**	800	1.05	0.98	1.12		693	1.05	0.97	1.13		686	1.27	1.17	1.37		446	0.97	0.88	1.07		103	1.05	0.86	1.29

Note 1: adjusted for adjusted for sex, age in 2005, level of education in 2005, marital status in 2005, country of birth, any mental disorder diagnosis before 2003, parental mental disorder diagnosis, and parental socioeconomic position during childhood.

Note 2: PER=precarious employment relationship; SSE= solo self-employment; HMJH=hybrid multiple job-holding; BO= business ownership; SER= standard employment relationship; SER/HI= standard employment relationship - high income; LQ=low quality; HQ=high quality

Table S8b. Fully adjusted hazard ratios for specific outcomes (depression, anxiety, stress related disorders, alcohol and non-alcohol use disorders) for women (n=1,275,850).

	Depression				Anxiety				Stress related disorders				Alcohol use disorders				Non-alcoholic use disorders			
	cases	HR2	CI95%		cases	HR2	CI95%		cases	HR2	CI95%		cases	HR2	CI95%		cases	HR2	CI95%	
**Constant SER**	14178	ref	ref		12142	ref	ref		10957	ref	ref		4449	ref	ref		1173	ref	ref	
**Constant SER/HI**	2532	0.76	0.72	0.79	1971	0.73	0.70	0.77	258	0.97	0.93	1.01	888	0.97	0.90	1.05	160	0.73	0.62	0.87
**Constant BO**	127	0.75	0.63	0.90	118	0.83	0.69	0.99	124	0.98	0.82	1.17	68	1.26	0.99	1.60	15	1.14	0.68	1.89
**Constant HMJH**	382	1.01	0.91	1.11	309	0.97	0.87	1.09	383	1.29	1.16	1.43	110	0.99	0.82	1.19	34	1.19	0.85	1.67
**Constant SSE**	412	0.89	0.81	0.98	391	1.00	0.90	1.11	358	1.06	0.95	1.17	175	1.14	0.98	1.32	46	1.23	0.92	1.66
**Constant PER**	1707	1.29	1.22	1.35	1556	1.26	1.20	1.34	1525	1.35	1.28	1.43	409	1.24	1.12	1.38	158	1.33	1.12	1.58
**Constant UE**	35	1.28	0.92	1.80	26	1.14	0.77	1.67	31	1.51	1.06	2.15	8	1.14	0.57	2.28	2	0.91	0.23	3.65
**Constant HQ**	1051	0.94	0.88	1.00	834	0.88	0.82	0.94	974	1.06	0.99	1.13	337	1.13	1.01	1.26	67	0.82	0.64	1.05
**Constant LQ**	282	1.38	1.23	1.56	229	1.24	1.09	1.42	230	1.39	1.21	1.58	86	1.55	1.25	1.92	31	1.67	1.16	2.40
**Fluctuating HQ**	469	0.91	0.83	1.00	413	0.96	0.87	1.06	431	1.03	0.94	1.14	144	1.04	0.88	1.23	43	1.14	0.84	1.55
**Fluctuating LQ**	33	1.36	0.97	1.92	23	1.08	0.72	1.62	30	1.57	1.09	2.24	11	1.67	0.92	3.02	4	1.82	0.68	4.88
**Fluctuating HQ and HMJH**	217	0.98	0.86	1.13	174	0.94	0.81	1.10	197	1.12	0.97	1.29	67	1.07	0.84	1.36	16	0.97	0.59	1.59
**Fluctuating LQ and HMJH**	119	1.07	0.89	1.28	113	1.16	0.97	1.40	120	1.34	1.12	1.60	39	1.23	0.89	1.71	6	0.66	0.29	1.47
**Fluctuating LQ and HQ**	2459	1.06	1.02	1.11	2168	1.05	1.00	1.10	2237	1.17	1.12	1.22	666	1.06	0.98	1.15	199	1.01	0.88	1.17
**Within HQ**	43	1.01	0.75	1.36	42	1.16	0.85	1.57	39	1.17	0.85	1.60	16	1.32	0.81	2.16	4	1.22	0.46	3.26
**Between HMJH and HQ**	307	0.96	0.85	1.07	247	0.91	0.80	1.03	314	1.22	1.09	1.36	85	0.94	0.76	1.17	14	0.57	0.34	0.97
**Between HMJH and LQ**	163	1.24	1.06	1.44	138	1.20	1.01	1.41	151	1.41	1.20	1.66	42	1.12	0.83	1.53	10	0.93	0.50	1.73
**Within LQ**	8	1.61	0.80	3.22	5	1.15	0.48	2.77	3	0.78	0.25	2.42	2	1.56	0.39	6.23	0	NA	NA	NA
**Upward LQ to HQ**	156	1.03	0.98	1.09	1402	1.04	0.98	1.10	1433	1.14	1.08	1.21	453	1.13	1.02	1.25	129	1.02	0.85	1.22
**Downward HQ to LQ**	1378	1.11	1.05	1.17	1262	1.15	1.09	1.22	1247	1.23	1.16	1.31	448	1.31	1.19	1.44	129	1.23	1.02	1.47
**Other**	311	1.02	0.91	1.14	302	1.12	1.00	1.26	345	1.37	1.23	1.53	87	1.05	0.84	1.30	29	1.16	0.80	1.68

Note 1: adjusted for adjusted for sex, age in 2005, level of education in 2005, marital status in 2005, country of birth, any mental disorder diagnosis before 2003, parental mental disorder diagnosis, and parental socioeconomic position during childhood.

Note 2: PER=precarious employment relationship; SSE= solo self-employment; HMJH=hybrid multiple job-holding; BO= business ownership; SER= standard employment relationship; SER/HI= standard employment relationship - high income; LQ=low quality; HQ=high quality

Table S8c. Fully adjusted hazard ratios for specific outcomes (depression, anxiety, stress related disorders, alcohol and non-alcohol use disorders) for men (n= 1,467,914).

	Depression				Anxiety				Stress related disorders				Alcohol use disorders				Non-alcoholic use disorders			
	cases	HR2	CI95%		cases	HR2	CI95%		cases	HR2	CI95%		cases	HR2	CI95%		cases	HR2	CI95%	
**Constant SER**	6576	ref	ref		5517	ref	ref		3929	ref	ref		6281	ref	ref		980	ref	ref	
**Constant SER/HI**	4409	0.85	0.80	0.90	3711	0.65	0.62	0.68	3163	0.76	0.72	0.80	4621	0.78	0.75	0.81	455	0.59	0.52	0.66
**Constant BO**	482	0.93	0.82	1.06	414	0.75	0.68	0.83	345	0.87	0.78	0.97	583	0.92	0.84	1.00	61	0.76	0.59	0.98
**Constant HMJH**	345	0.95	0.81	1.13	258	0.80	0.70	0.91	241	1.04	0.91	1.18	278	0.81	0.72	0.91	37	0.79	0.57	1.10
**Constant SSE**	913	0.97	0.87	1.07	691	0.82	0.76	0.89	548	0.91	0.83	0.99	954	0.91	0.85	0.97	106	0.84	0.69	1.03
**Constant PER**	1259	1.28	1.17	1.41	1101	1.14	1.07	1.22	869	1.22	1.13	1.31	1009	1.23	1.15	1.32	294	1.45	1.27	1.66
**Constant UE**	68	1.30	0.86	1.95	52	1.25	0.95	1.65	47	1.55	1.16	2.07	82	1.88	1.51	2.35	23	2.86	1.89	4.33
**Constant HQ**	121	1.00	0.91	1.10	1019	0.84	0.79	0.90	862	0.97	0.90	1.04	108	0.89	0.83	0.95	160	0.79	0.67	0.93
**Constant LQ**	322	1.45	1.22	1.71	257	1.15	1.01	1.30	223	1.32	1.15	1.51	325	1.56	1.39	1.74	79	1.72	1.36	2.17
**Fluctuating HQ**	564	0.93	0.82	1.06	479	0.82	0.75	0.90	378	0.89	0.80	0.99	578	0.94	0.86	1.03	93	0.95	0.77	1.18
**Fluctuating LQ**	39	1.37	0.85	2.22	41	1.40	1.02	1.91	34	1.52	1.08	2.14	39	1.41	1.03	1.94	21	3.17	2.05	4.90
**Fluctuating HQ and HMJH**	246	0.79	0.70	0.90	184	0.73	0.63	0.84	181	1.03	0.97	1.09	185	0.72	0.62	0.84	31	0.82	0.57	1.17
**Fluctuating LQ and HMJH**	181	1.21	0.97	1.51	139	0.97	0.82	1.15	140	1.30	1.09	1.54	149	1.05	0.89	1.24	32	1.26	0.88	1.80
**Fluctuating LQ and HQ**	2181	0.96	0.91	1.01	1836	0.92	0.88	0.97	1517	1.04	0.98	1.10	1917	1.02	0.96	1.07	410	1.12	0.99	1.26
**Within HQ**	44	0.92	0.58	1.46	44	1.07	0.79	1.43	35	1.18	0.84	1.64	50	1.16	0.88	1.53	7	0.97	0.46	2.05
**Between HMJH and HQ**	246	0.99	0.83	1.17	184	0.73	0.64	0.83	181	0.99	0.87	1.13	185	0.77	0.68	0.88	37	0.75	0.54	1.05
**Between HMJH and LQ**	184	1.01	0.80	1.28	138	0.92	0.78	1.09	117	1.05	0.87	1.26	136	0.91	0.77	1.09	30	1.18	0.82	1.70
**Within LQ**	13	1.01	0.33	3.14	11	1.64	0.91	2.96	14	2.77	1.64	4.69	11	1.75	0.97	3.17	2	1.53	0.38	6.15
**Upward LQ to HQ**	1144	1.02	0.93	1.13	1004	0.94	0.88	1.01	831	1.06	0.98	1.14	973	1.00	0.93	1.07	199	0.98	0.84	1.15
**Downward HQ to LQ**	1212	1.01	0.92	1.12	999	0.96	0.89	1.02	780	1.02	0.94	1.10	1158	1.13	1.06	1.21	218	1.14	0.99	1.33
**Other**	494	0.98	0.85	1.14	385	0.94	0.84	1.04	348	1.12	1.01	1.26	343	0.89	0.80	0.99	75	1.04	0.82	1.32

Note: adjusted for adjusted for sex, age in 2005, level of education in 2005, marital status in 2005, country of birth, any mental disorder diagnosis before 2003, parental mental disorder diagnosis, and parental socioeconomic position during childhood.

Note 2: PER=precarious employment relationship; SSE= solo self-employment; HMJH=hybrid multiple job-holding; BO= business ownership; SER= standard employment relationship; SER/HI= standard employment relationship - high income; LQ=low quality; HQ=high quality

Figure S1. Flow chart of the (total) study population

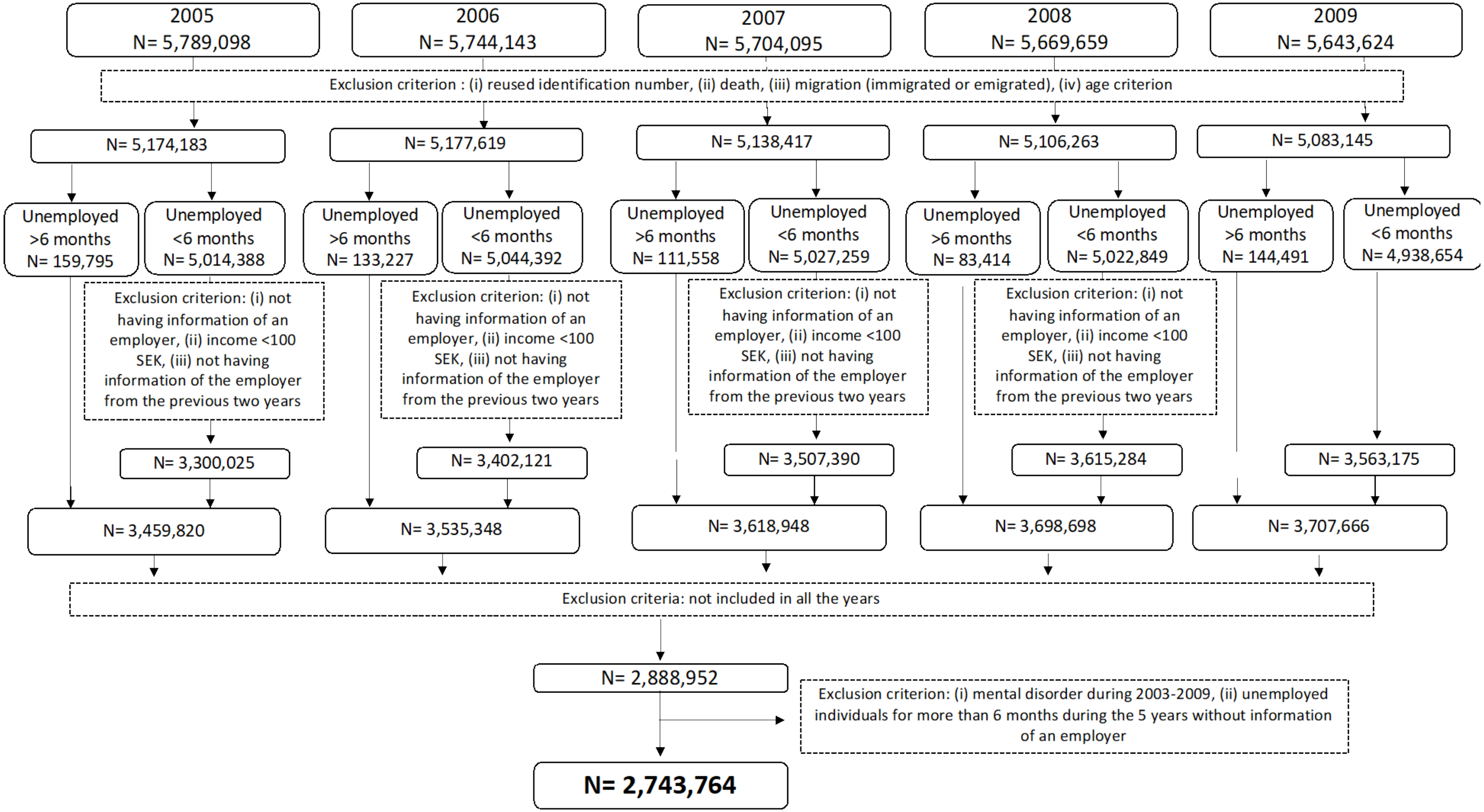

Figure S2. Directed Acyclic Graph (DAG) for the effect of employment trajectories on mental health.

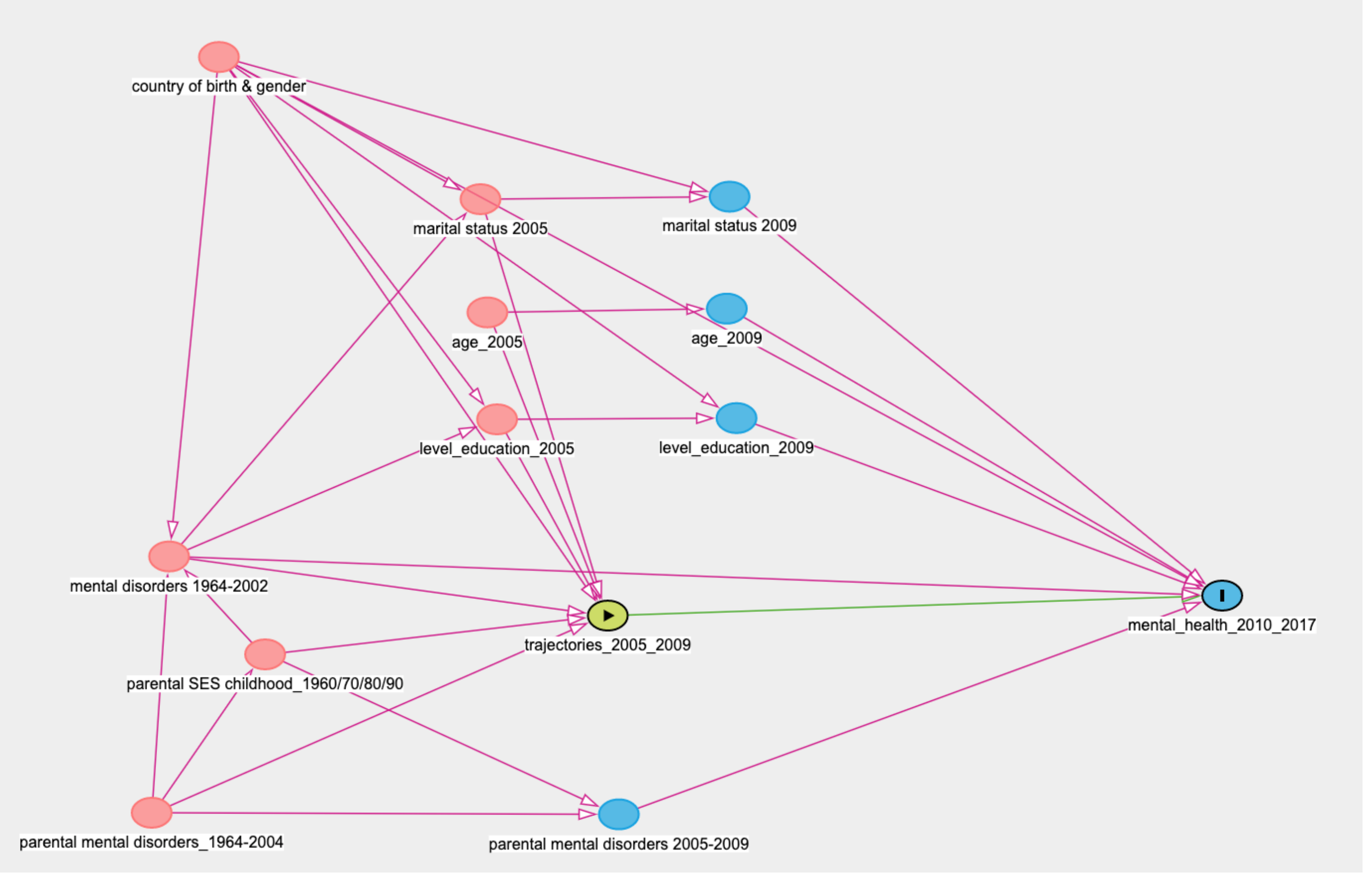

The minimal sufficient adjustment sets for estimating the total effect of the low-quality employment trajectories on mental health (mental disorders and/or suicide attempt) are several, and for this paper we have chosen the following one: age, country of birth, gender, level of education, marital status, mental disorder (diagnosis) occurring before 2003 (please note that individuals with a diagnose of any mental disorder during 2003-2009 are excluded from the study population), parental socioeconomic position during the childhood of individuals, any parental mental disorder (diagnosis) occurring before exposure measurement.

Textor J, van der Zander B, Gilthorpe MS, Liśkiewicz M, Ellison GTH. Robust causal inference using directed acyclic graphs: the R package 'dagitty.' Int J Epidemiol. 2017 Jan 15;dyw341
